# Bone marrow-derived CD169^+^ macrophages promote autoimmune hepatitis by recruiting CCR2^+^ monocytes via secreting CCL12

**DOI:** 10.1038/s12276-025-01607-w

**Published:** 2025-12-22

**Authors:** Bingru Lin, Huayang Zhang, Pengwei Zhu, Jianing Chen, Dingwu Li, Jiaming Zhou, Tiantian Zhang, Qingxia Chen, Chenxi Tang, Xin Song, Hang Zeng, Jinghua Wang, Jie Zhang, Zhengrui You, Xiong Ma, Chaohui Yu

**Affiliations:** 1 Department of Gastroenterology, Zhejiang Provincial Clinical Research Center for Digestive Diseases, The First Affiliated Hospital, Zhejiang University School of Medicine, Hangzhou, China; 2 Division of Gastroenterology and Hepatology, Key Laboratory of Gastroenterology and Hepatology, Ministry of Health, State Key Laboratory for Oncogenes and Related Genes, Renji Hospital, School of Medicine, Shanghai Jiao Tong University, Shanghai, China; Shanghai Institute of Digestive Disease, Shanghai, China; 3 Departments of Cardiology, The First Affiliated Hospital, Zhejiang University School of Medicine, Hangzhou, China; 4 State Key Laboratory for Diagnosis and Treatment of Infectious Diseases, National Clinical Research Center for Infectious Diseases, Collaborative Innovation Center for Diagnosis and Treatment of Infectious Diseases, The First Affiliated Hospital, School of Medicine, Hangzhou, China

**Keywords:** Autoimmune hepatitis, Acute inflammation, Experimental models of disease, Autoimmune diseases

## Abstract

CD169^+^ macrophages, a unique subset of macrophages that cannot be simply defined as M1 or M2 macrophages, have been reported to be associated with various autoimmune diseases. However, the role of CD169^+^ macrophages in autoimmune hepatitis (AIH) is largely unknown. Here we found that the infiltration of CD169^+^ macrophages increased in the liver of patients with AIH and strongly positively correlated with inflammation degree. In a mouse model, depletion of CD169^+^ macrophages ameliorated ConA-induced acute liver injury. Immune homeostasis was also improved when CD169^+^ macrophages were depleted, as the infiltration of monocytes, macrophages and T cells decreased. Bone marrow-derived Ly6C^hi^CD169^+^ macrophages were further identified as the crucial subset in AIH. Next, we found that CD169^+^ macrophages were IFNγ-responsive and IFNγ could induce the expression of CD169. In response to the IFNγ signal, CD169^+^ macrophages actively secrete chemokine (C–C motif) ligand (CCL12), thus recruiting CCR2^+^ monocytes and macrophages to exacerbate AIH. Finally, neutralizing CCL12 improved AIH. Our results suggest that bone marrow-derived CD169^+^ macrophages, the key subset of macrophages in AIH, actively secrete CCL12 in response to IFNγ to recruit CCR2^+^ monocytes and macrophages, thus exacerbating AIH. The CD169^+^ macrophages are a potential therapeutic target in AIH.

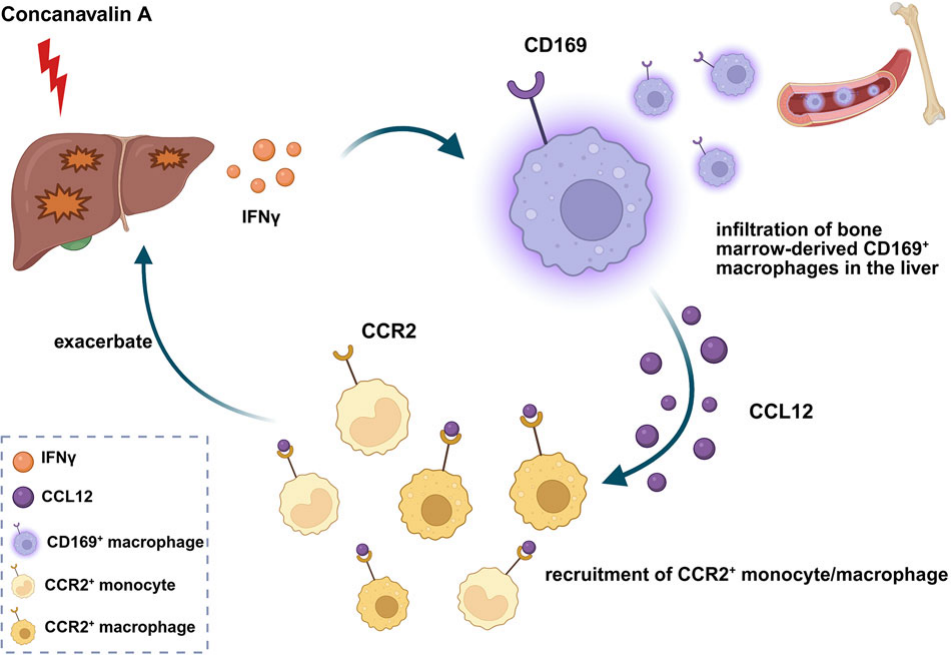

## CD169+ Macrophages Drive Inflammation in Autoimmune Hepatitis

Autoimmune hepatitis (AIH) is a liver disease in which the immune system attacks liver cells. Researchers wanted to understand the role of a specific type of immune cell, CD169^+^ macrophages. The study involved analyzing liver tissues from 40 patients with AIH and conducting experiments on mice. They found that CD169^+^ macrophages increased in AIH and were linked to disease severity. In mice, removing these cells reduced liver damage. The study showed that bone marrow-derived CD169^+^ macrophages, not those from the liver itself, were crucial in worsening AIH. These cells produce a protein called CCL12, which attracts more immune cells to the liver. The researchers concluded that targeting these specific macrophages could be a new way to treat AIH. In the future, therapies could focus on reducing the activity of these cells to help manage or prevent AIH progression.

This summary was initially drafted using artificial intelligence, then revised and fact-checked by the author.

## Introduction

Autoimmune hepatitis (AIH) is an obscure inflammatory liver disease that may be caused by the interaction of individual genetic susceptibility and environmental factors^1,2^. It is characterized by immune-mediated destruction of hepatocytes leading to progressive necrotizing inflammation of the liver^2–4^. AIH is a disease of the hepatic parenchyma that can manifest in both acute and chronic forms. The clinical presentation often resembles acute hepatitis and may progress to liver cirrhosis and end-stage liver failure^1,2^. ConA-induced acute liver injury animal model is widely used to mimic clinical acute hepatitis and immune-mediated liver injury in humans^4–7^.

Studies have shown that macrophages in the liver play an important role in ConA-induced AIH^8,9^. Macrophages are the most abundant immune cells in the liver and are involved in maintaining liver immune homeostasis^10,11^. However, macrophages in the liver exhibit substantial heterogeneity. Kupffer cells (KCs) are the predominant macrophage population in the healthy liver, and they originate from the yolk sac^12^. KCs replenish relying on self-renewal under homeostatic conditions, independent of bone marrow-derived macrophages (BMDMs)^13^. When hepatitis occurs, KCs are activated and then inflammatory Ly6C^hi^ monocyte-derived macrophages (MoMFs) are recruited into the liver to participate in the inflammatory response. Blocking the recruitment of MoMFs reduces hepatic inflammation and fibrosis^14^. Although each macrophage subset plays a distinct role in maintaining liver homeostasis, the concrete cellular and molecular mechanisms of different subsets in regulating liver immunity remain largely unknown.

CD169, also known as sialoadhesin or siglec1, is a prototype member of the siglec family of sialic acid-binding immunoglobulin-like lectin^15^. It is composed of 17 immunoglobin-like domains and a short cytoplasmic tail that lacks signaling motifs^16^. CD169 is initially reported as a marker for a macrophage subset isolated from bone marrow, lymph nodes, liver and spleen^17^. In recent years, CD169^+^ macrophages have been considered to be a distinct group of macrophages that cannot be simply defined as M1 or M2 macrophages^18^. For now, CD169^+^ macrophages have been shown to play an important role in various autoimmune diseases. It has been reported that CD169^+^ macrophages increased in multiple sclerosis and experimental autoimmune encephalitis^19^. Besides, the relative number of peripheral CD169^+^ monocytes is significantly and positively correlated with disease activity in rheumatoid arthritis and may even serve as a biomarker^20,21^. Similarly, CD14^+^CD169^+^ monocytes were significantly increased in patients with systemic lupus erythematosus^22^ and children with type 1 diabetes^23^. In addition, CD169 is highly expressed on peripheral blood monocytes in patients with primary biliary cholangitis (PBC)^24^. AIH and PBC are both classified as autoimmune liver diseases. However, the relationship between CD169^+^ macrophages and AIH is still largely unknown.

CD169-diphtheria toxin receptor (DTR) mice express DTR at the CD169 gene locus through genetic engineering techniques, enabling researchers to specifically eliminate cells expressing CD169 by injecting diphtheria toxin (DT)^25^.

Here, our study revealed that the number of CD169^+^ macrophages is positively correlated with disease severity in patients with AIH. In mice, depletion of CD169^+^ macrophages alleviated ConA-induced liver injury and necrosis, and improved immune homeostasis in the liver. Rather than liver-resident CD169^+^ macrophages, bone marrow-derived CD169^+^ macrophages macrophages were shown to be the key subset in AIH. Compared with CD169^−^ macrophages, CD169^+^ macrophages selectively secrete CCL12 to recruit CCR2^+^ monocytes and macrophages to further exacerbate the acute liver injury in response to IFNγ signal.

## Materials and methods

### Human participants

Liver tissues from 40 patients with AIH, 21 patients with PBC, 12 patients with metabolic dysfunction-associated steatotic liver disease (MASLD) and 7 patients with chronic hepatitis B (CHB) were obtained from ultrasound-guided needle liver biopsies at diagnosis. Liver tissues of six healthy controls (HCs) were collected from donor livers before transplantation. Demographic and clinical characteristics of the study participants are presented in Supplementary Table 1. All individuals were enrolled in Shanghai Renji Hospital and provided written informed consent. The study was carried out under the principles of the Declarations of Helsinki and Istanbul and approved by the Shanghai Jiao Tong University Ethics Committee.

### Mice

C57BL/6J mice purchased from Zhejiang Academy of Medical Sciences, aged 8–12 weeks, were used in the current study. CD169-DTR mice (C57BL/6J background) were gifted by Dr. Jiong Chen (Ningbo University, China). The animal study was approved by the Animal Care and Use Committee of the First Affiliated Hospital, Zhejiang University School of Medicine. Additional materials and methods can be found in the Supplementary Information.

## Results

### CD169^+^ macrophages were significantly increased in the livers of patients with AIH and positively correlated with disease severity

**Fig. 1:**
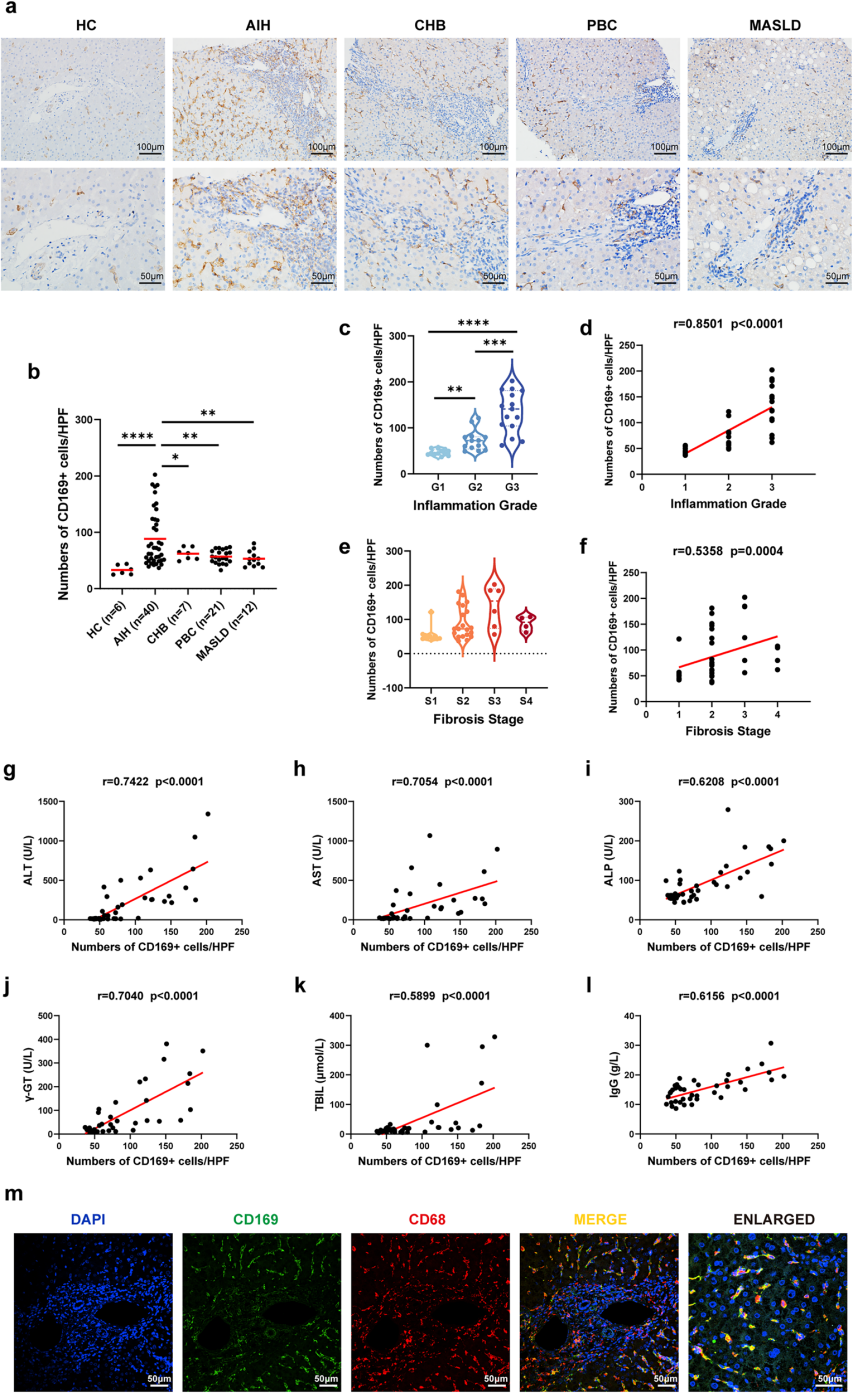
CD169^+^ macrophages increased in the liver of patients with AIH. **a** Immunohistochemical staining images of CD169^+^ macrophages in the liver of HCs and patients with AIH, CHB, PBC and MASLD. **b** The number of CD169^+^ macrophages in the liver per HPF. **c** The number of CD169^+^ macrophages in different inflammation grades. **d** The correlation of the number of CD169^+^ macrophages and different inflammation grades. **e** The number of CD169^+^ macrophages in different fibrosis stages. **f** The correlation of the number of CD169^+^ macrophages and different fibrosis stages. **g**–**l** The correlation of the number of CD169^+^ macrophages and ALT (**g**), AST (**h**), ALP (**i**), γ-GT (**j**), TBIL (**k**) and IgG (**l**) levels. **m** Costaining immunofluorescent images of CD169 and CD68 in the liver of patients with AIH. Results are expressed as mean ± standard error. *P < 0.05, **P < 0.01, ***P < 0.001, ****P < 0.0001.

First, we confirmed that CD169^+^ cells in human liver are macrophages by analyzing the human liver single-cell RNA sequencing (scRNA-seq) dataset GSE124395 (Supplementary Fig. 1a–c). To clarify the association between CD169^+^ macrophages and AIH, we first analyzed the amount of CD169^+^ macrophages in the liver of HCs (*n* = 6) and patients with AIH (*n* = 40), CHB (*n* = 7), PBC (*n* = 21) and MASLD (*n* = 12). The immunohistochemical staining results of CD169 indicated a significant increase in the number of CD169^+^ macrophages in the livers of patients with AIH compared with HCs and patients with other liver diseases (Fig. 1a, b).

Next, we found that the numbers of CD169^+^ macrophages in the liver increased with the progression of inflammation grade (Fig. 1c) and had a very strong positive correlation (*r* = 0.8501, *P* < 0.0001) with liver inflammation grade (Fig. 1d). As for the fibrosis stage, no statistical significance was observed among the different fibrosis stages (Fig. 1e). A moderate correlation (*r* = 0.5358, *P* = 0.0004) was observed between the fibrosis stage and the number of CD169^+^ macrophages in the 40 patients with AIH enrolled in the study (Fig. 1f). To further investigate the potential clinical importance of CD169^+^ macrophages in AIH, we analyzed the relationship between several biochemical parameters and the number of CD169^+^ macrophages in AIH. Surprisingly, the number of CD169^+^ macrophages in AIH was significantly positively correlated with alanine aminotransferase (ALT) (*r* = 0.7422, *P* < 0.0001), aspartate transaminase (AST) (*r* = 0.7054, *P* < 0.0001), alkaline phosphatase (ALP) (*r* = 0.6028, *P* < 0.0001), γ-glutamyl transferase (γ-GT) (*r* = 0.7040, *P* < 0.0001), total bilirubin (TBIL) (*r* = 0.5899, *P* < 0.0001) and immunoglobulin G (IgG) (*r* = 0.6156, *P* < 0.0001) (Fig. 1g–l). Consistent with previous studies^26,27^, our results also suggest that CD169^+^ cells in the liver of patients with AIH are macrophages as they costain with the macrophage marker CD68 (Fig. 1m). These results together suggest that CD169^+^ macrophages may play an important role in AIH, especially in the progression of inflammation.

### The increased CD169^+^ cells in the liver of mice with AIH are mainly CD11b^+^F4/80^+^Ly6C^+^ infiltrating macrophages

**Fig. 2:**
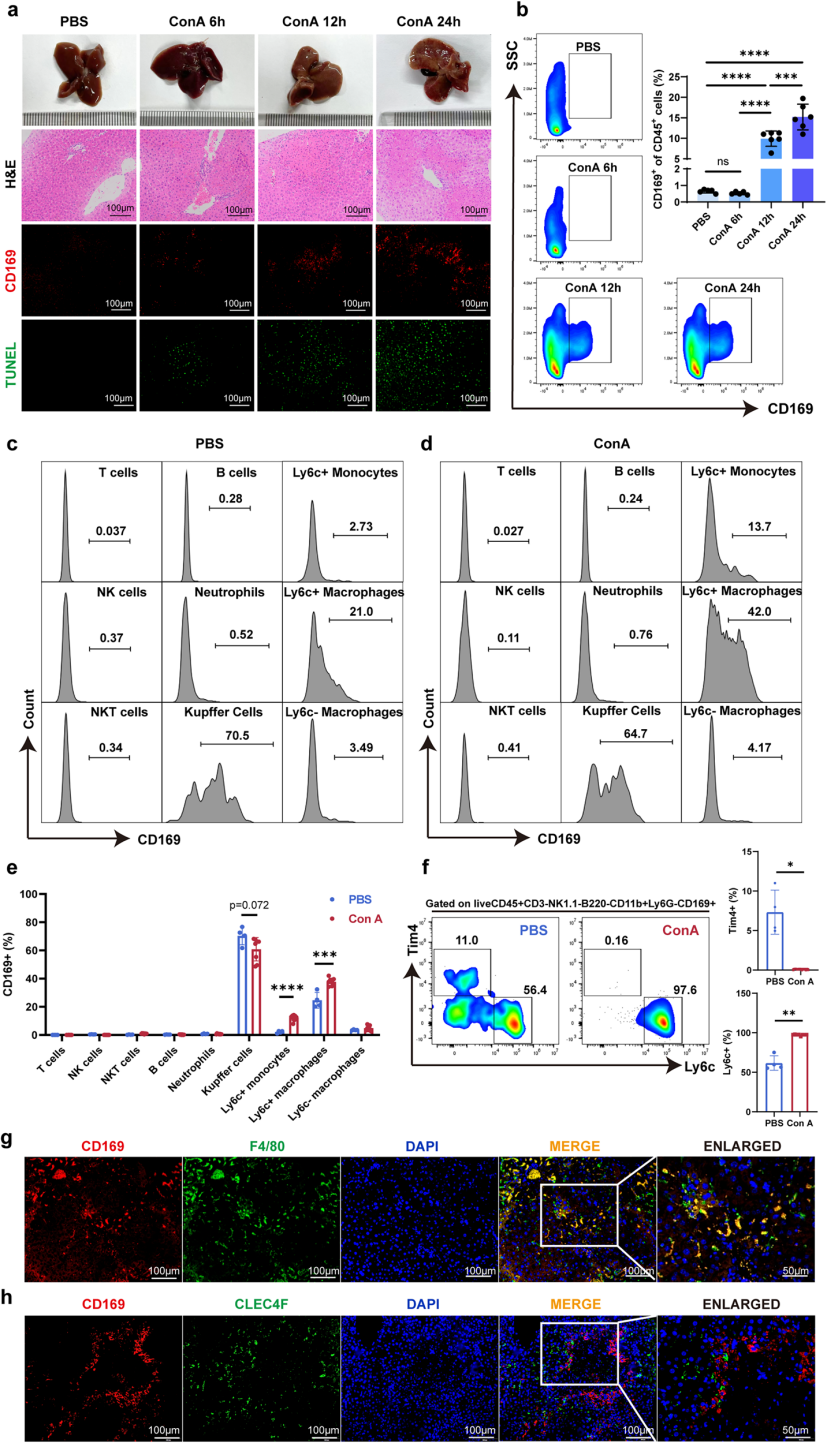
CD11b^+^F4/80^+^Ly6C^+^CD169^+^ macrophages increased in the liver of mice administrated with ConA. **a** Macroscopic images, H&E staining images, immunofluorescent images of CD169^+^ macrophages, and TUNEL staining images at indicated time points after ConA administration. **b** Frequency of CD169^+^ cells in the CD45^+^ cells. **c** CD169^+^ proportions in various immune cells after 12 h injected with PBS. **d** CD169^+^ proportions in various immune cells after 12 h injected with 10 mg/kg ConA. **e** CD169^+^ proportions in various immune cells after 12 h injected with PBS or 10 mg/kg ConA. **f** Tim4^+^ and Ly6c^+^ proportions in CD45^+^CD3^−^NK1.1^−^B220^−^CD11b^+^Ly6G^−^CD169^+^ cell population after 12 h injected with PBS or 10 mg/kg ConA. **g** Costaining immunofluorescent images of F4/80 and CD169 after 12 h injected with PBS or 10 mg/kg ConA. **h** Costaining immunofluorescent images of CLEC4F and CD169 after 12 h injected with PBS or 10 mg/kg ConA. Results are expressed as mean ± standard error. *P < 0.05, **P < 0.01, ***P < 0.001, ****P < 0.0001.

To further illustrate the relationship between CD169^+^ macrophages and the progression of inflammation in AIH, we conducted time gradient AIH modeling on wild-type (WT) mice. After ConA administration for 0, 6, 12 and 24 h, as liver inflammation intensifies and the necrotic area expands, the amount of CD169^+^ macrophages in the liver also gradually increases (Fig. 2a and Supplementary Fig. 2a). Consistent with these results, the proportion of CD169^+^ cells in CD45^+^ cells increased after 12 h of modeling and further increased at 24 h (Fig. 2b). To further investigate the phenotypes of CD169^+^ cells in the liver, multicolor flow cytometry staining on nonparenchymal liver cells was performed. We classified T cells, natural killer (NK) cells, natural killer T (NKT) cells, B cells, neutrophils, KCs, Ly6C^+^ monocytes, Ly6C^+^ macrophages and Ly6C^−^ macrophages (Supplementary Fig. 2b) and then counted the proportion of CD169^+^ cells in each of these subgroups. In the PBS group, all macrophage populations expressed CD169, with the highest proportion of KC populations (Fig. 2c). After 12 h of ConA administration, the proportion of CD169^+^ cells increased in the Ly6C^+^ monocytes group and Ly6C^+^ macrophages group (Fig. 2d, e), indicating that the increased CD169^+^ cells were possibly MoMFs. Ly6C^+^ monocytes and macrophages also increased after ConA administration (Supplementary Fig. 2c). TIM4 is a marker for liver-resident KCs^28,29^. We observed that, after ConA administration, TIM4^+^CD169^+^ proportions decreased significantly while Ly6C^+^CD169^+^ proportions increased (Fig. 2f). Immunofluorescence results further confirmed that the increased CD169^+^ cells in the AIH liver are MoMFs but not KCs. As shown in Fig. 2g, h, almost all CD169 showed costaining with F4/80, whereas most CD169 did not costain with CLEC4F, another marker for KCs^28^.

In summary, CD169^+^ cells increased in the liver of AIH mice, and most of the increased CD169^+^ cells are CD11b^+^F4/80^+^Ly6C^+^ infiltrating macrophages.

### CD169^+^ macrophages were required for ConA-induced hepatic necrosis

**Fig. 3:**
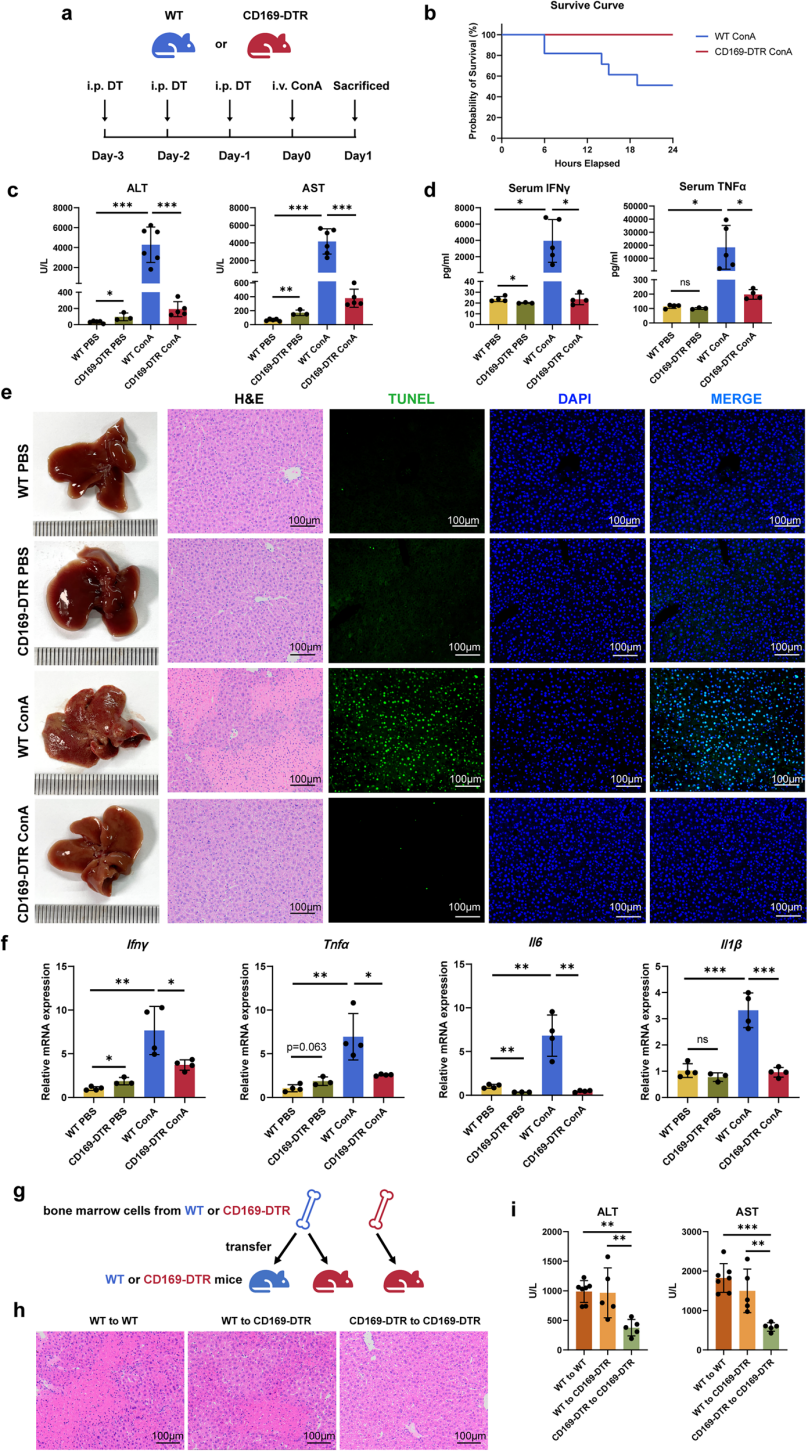
CD169^+^ macrophage depletion ameliorated ConA-induced AIH. **a** Schematic diagram of CD169^+^ cell depletion and AIH modeling. **b** Survival curves within 24 h after 20 mg/kg ConA administration in WT or CD169-DTR mice. **c** Serum ALT and AST levels at 24 h after 20 mg/kg ConA administration. Serum IFNγ and TNFα levels at 24 h after 20 mg/kg ConA administration. Macroscopic images, H&E staining images and TUNEL staining images at 24 h after 20 mg/kg ConA administration. Relative IFNγ, TNFα, IL6 and IL1β mRNA levels at 24 h after 20 mg/kg ConA administration. **d** Schematic diagram of bone marrow transplantation and H&E staining images at 24 h after 20 mg/kg ConA administration. **e** H&E staining images in the bone marrow-transplanted groups at 24 h after 20 mg/kg ConA administration. **f** Serum ALT and AST levels in the bone marrow-transplanted groups at 24 h after 20 mg/kg ConA administration. Results are expressed as mean ± standard error. *P < 0.05, **P < 0.01, ***P < 0.001.

To further elucidate the role of CD169^+^ macrophages in AIH, we administered DT into CD169-DTR mice to clear CD169^+^ macrophages^25^. The depletion of CD169^+^ macrophages was verified through flow cytometry analysis (Supplementary Fig. 3a) and immunohistochemistry staining (Supplementary Fig. 3b). An equivalent dose of DT was also injected into WT mice to control for any potential effects caused by DT injection (Fig. 3a). A lethal dose of 20 mg/kg of ConA was injected into WT and CD169-DTR mice, and the mortality of the mice was recorded. Surprisingly, 5 out of 11 WT mice died while all CD169-DTR mice survived 24 h after ConA administration (Fig. 3b). Compared with the WT ConA group, the levels of serum ALT and AST significantly reduced in the CD169-DTR ConA group (Fig. 3c). Besides, serum IFNγ and TNFα levels, the main inflammatory cytokines that cause liver necrosis and infiltration of leukocytes^30^, were also lower in the CD169-DTR ConA group than those in the WT ConA group (Fig. 3d). Following the depletion of CD169^+^ macrophages, a notable improvement in liver injury and necrosis was observed, as indicated by visual observation, hematoxylin and eosin (H&E) staining and terminal deoxynucleotidyl transferase dUTP nick-end labeling (TUNEL) staining (Fig. 3e). Notably, no hepatic necrosis was observed microscopically on H&E staining in the CD169-DTR ConA group. In addition, quantitative reverse transcription polymerase chain reaction (qRT–PCR) results showed that the expression of IFNγ, TNFα, IL6 and IL1β decreased when CD169^+^ macrophages were depleted, indicating alleviated liver inflammation in the CD169-DTR ConA group (Fig. 3f).

Taken together, our results emphasize the critical role of CD169^+^ macrophages in promoting ConA-induced hepatitis.

### Bone marrow-derived CD169^+^ macrophages are a key subset in AIH progression

According to our present results, there are at least two distinct populations of CD169^+^ macrophages in the liver: one is yolk sac-derived CD11b^+^F4/80^+^TIM4^+^CD169^+^ KCs, and the other is bone marrow-derived CD11b^+^F4/80^+^Ly6C^+^CD169^+^ infiltrating macrophages. To clarify which origin of CD169^+^ macrophages plays a more critical role in AIH, we performed bone marrow transplantation (Fig. 3g). When WT bone marrow cells were transplanted into CD169-DTR mice and DT was applied, the bone marrow-derived WT CD169^+^ cells remained, while the liver resident CD169^+^ KCs of CD169-DTR mice were depleted. The efficacy of bone marrow transplantation was confirmed by genotyping (Supplementary Fig. 3c). We found that chimeric CD169-DTR mice with WT bone marrow exhibited a phenotype similar to WT mice after ConA administration, as they showed more severe liver damage and necrosis compared with CD169-DTR mice transplanted with CD169-DTR bone marrow (Fig. 3h), with higher serum ALT and AST levels (Fig. 3i).

Together, our results suggest that the bone marrow-derived CD169^+^ macrophages are the vital subset of macrophages in ConA-induced liver injury.

### CD169^+^ macrophage depletion altered the immune microenvironment in AIH

**Fig. 4:**
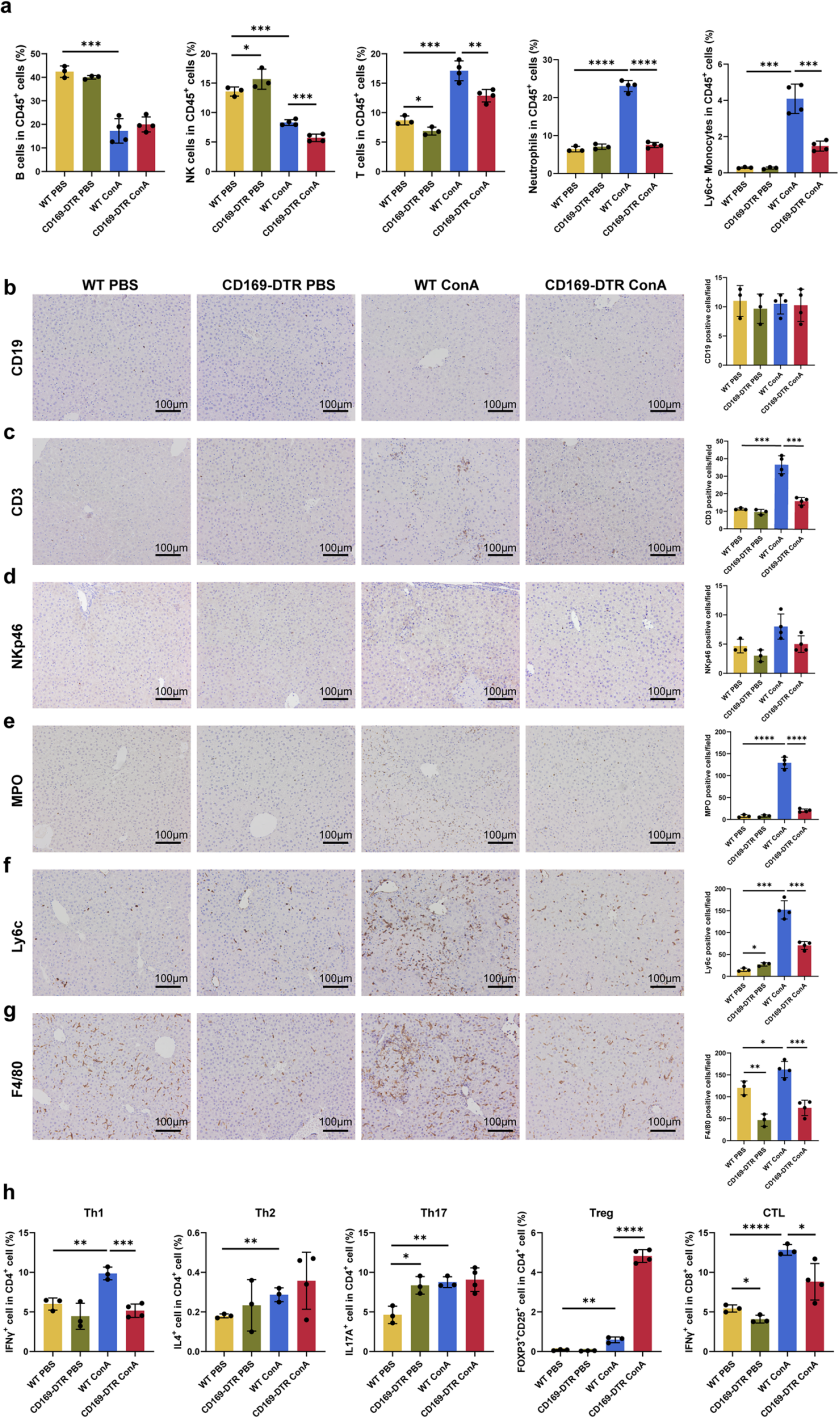
The immune microenvironment was altered following CD169^+^ macrophage depletion. **a** The frequency of B cells, NK cells, T cells, neutrophils and Ly6C^+^ monocytes in CD45^+^ cells. **b**–**g** Immunohistochemical staining and quantification of CD19 (**b**), CD3 (**c**), NKp46 (**d**), MPO (**e**), Ly6C (**f**) and F4/80 (**g**) positive cells. **h**, The frequency of Th1, Th2, Th17, T_reg_ and CTL. Results are expressed as mean ± standard error. *P < 0.05, **P < 0.01, ***P < 0.001, ****P < 0.0001.

Next, to further investigate the effects of CD169^+^ macrophage depletion on liver immune homeostasis, we performed multicolor flow cytometry to examine the alterations in various immune cells. The gating strategy is shown in Supplementary Fig. 4a. We found that, in the ConA groups, the frequency of NK cells, T cells, neutrophils and Ly6C^+^ monocytes decreased (Fig. 4a and Supplementary Fig. 4b), while the proportion of macrophages increased relatively after CD169^+^ macrophage depletion (Supplementary Fig. 4c). Similarly, immunohistochemistry results showed that, following clearing of CD169^+^ macrophages, the absolute numbers of T cells (identified by CD3), neutrophils (identified by MPO), monocytes (identified by LY6C) and macrophages (identified by F4/80) significantly decreased in the ConA groups, while B cells (identified by CD19) and NK cells (identified by NKp46) did not show any difference (Fig. 4b–g).

Considering the importance of T cells in AIH, we further performed flow cytometry to analyze their activation (Supplementary Fig. 5a) and differentiation (Supplementary Fig. 6a). First, the proportion of CD4^+^ T cells decreased after depleting CD169^+^ macrophages, while the proportion of CD8^+^ T cells increased (Supplementary Fig. 5b). Our results suggest that, compared with the WT ConA group, the CD44^−^CD62L^+^ naive CD4^+^ T cell frequency did not change in the CD169-DTR ConA group (Supplementary Fig. 5c), while the proportion of CD25^+^ activated CD4^+^ T cells showed a minor decrease (Supplementary Fig. 5d). The proportion of naive CD8^+^ T cells decreased in the CD169-DTR ConA group (Supplementary Fig. 5e), while the proportion of activated CD8^+^ T cells remained unchanged (Supplementary Fig. 5f). As for the differentiation of T cells, CD169^+^ macrophage depletion inhibited the differentiation of CD4^+^ T cells toward Th1 (identified by IFNγ^+^), promoted the differentiation of T_reg_ (identified by FOXP3 ^+^CD25^+^) and had no significant effect on Th2 (identified by IL4^+^) and Th17 (identified by IL17A^+^). Besides, the proportion of IFNγ-expressing CD8^+^ cytotoxic T lymphocytes (CTL) significantly decreased after CD169^+^ macrophage depletion (Fig. 4h and Supplementary Fig. 6b–f).

Collectively, the elimination of CD169 macrophages reduced the number of various pro-inflammatory immune cells in the AIH liver and ameliorated the inflammatory state of the AIH liver.

### CD169^+^ macrophages are IFNγ responsive

**Fig. 5:**
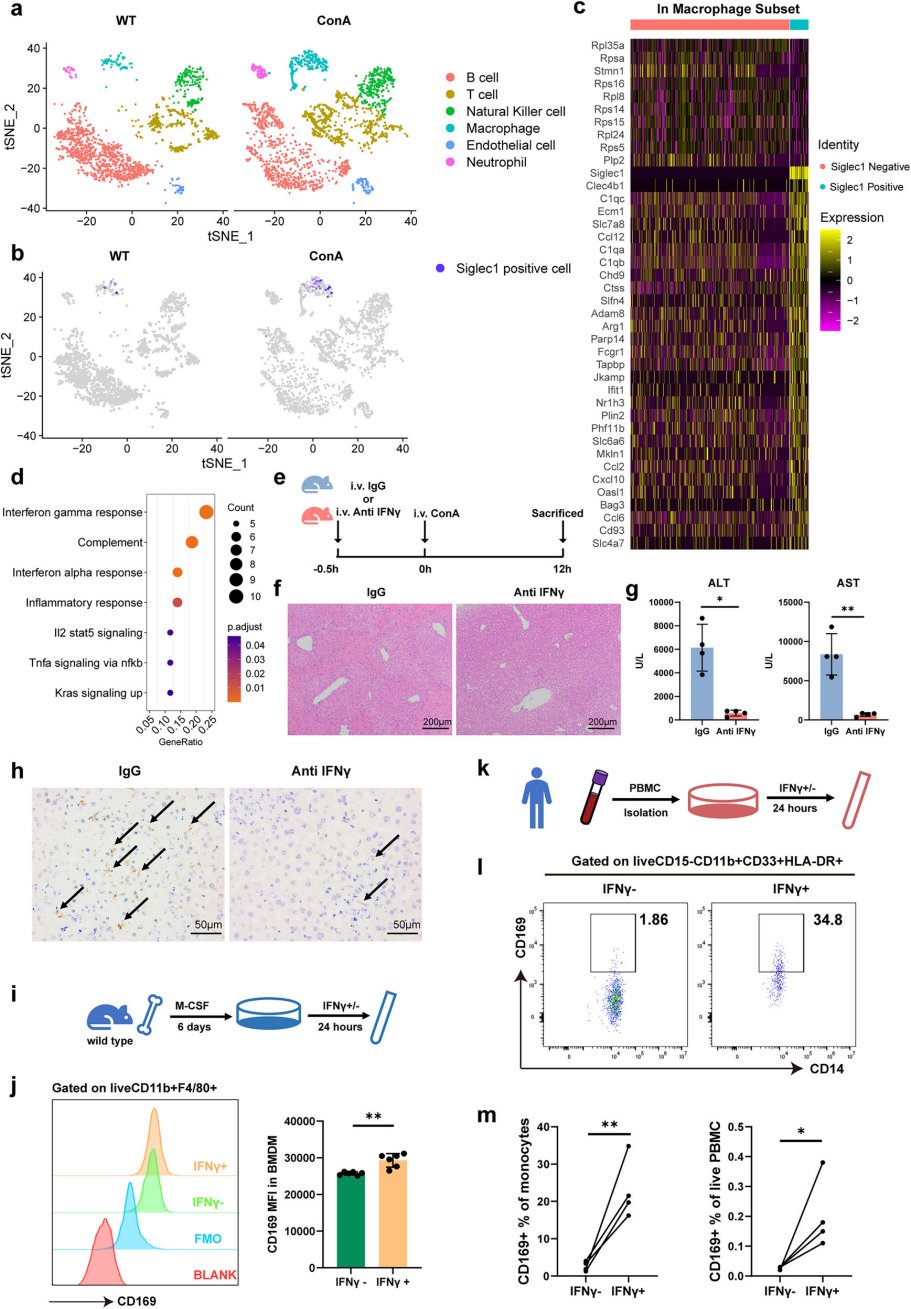
Single-cell sequence analysis revealed that CD169^+^ macrophages are IFNγ responsive. **a** Identification of cell clusters from the GSE201006 dataset. **b** Expression of siglec1 in the different subsets. **c** Differentially expressed genes in siglec1-positive and siglec1-negative macrophages. **d** HALLMARK analysis of the upregulated genes enriched in the siglec1-positive macrophage subset. **e** Schematic diagram of IFNγ neutralization. Anti-mouse IFNγ antibody and isotype control were both used at 25 μg/g bodyweight intravenously. To induce AIH, 10 mg/kg ConA was injected into mice intravenously. **f** H&E staining images of the AIH mice treated with IFNγ neutralizing antibody or isotype antibody. **g** Serum ALT and AST levels. **h** Immunohistochemical staining of CD169. Arrows indicate positive staining. **i** Schematic diagram of mouse BMDMs stimulated by IFNγ. **j** Measurement of CD169 mean fluorescence intensity in the BMDMs with or without stimulation with IFNγ in vitro. **k** Schematic diagram of human PBMCs stimulated by IFNγ. **l** Representative plots of CD14^+^CD169^+^ monocytes in human PBMCs with or without stimulation with IFNγ in vitro. **m** The proportion of CD14^+^CD169^+^ monocytes in the total monocyte group or the total live PBMC group. Results are expressed as mean ± standard error. *P < 0.05, **P < 0.01.

To further identify the role of CD169^+^ macrophages in AIH, the scRNA-seq dataset GSE201006 involving ConA-induced AIH in mice was analyzed. As expected, *siglec1*, the gene encoding CD169, was predominantly expressed in the macrophage population with or without ConA exposure (Fig. 5a). Also, the number of siglec1-expressing cells in the ConA group was higher than that in the WT group (Fig. 5b). To further clarify the differences between CD169^+^ macrophages and CD169^−^ macrophages, we classified macrophages in the ConA group according to their *siglec1* expression (Fig. 5c). Compared with CD169^−^ macrophages, CD169^+^ macrophages express higher levels of *Clec4b1*, *C1qc*, *Ecm1*, *Slc7a8*, *Ccl12* and so on (Fig. 5c). According to the HALLMARK enrichment analysis, CD169^+^ macrophages had higher expression of IFNγ-responsive genes (Fig. 5d), including *Parp14*, *Fcgr1*, *Tapbp*, *Ccl2*, *Cxcl10*, *Oasl1*, *Nampt*, *Cmpk2*, *Trim26* and *Lap3*. These findings align with prior results demonstrating concurrent elevation of IFNγ levels and an increased proportion of CD169⁺ macrophages in AIH (Figs. 2a and 3f). Next, after neutralizing IFNγ in mice (Fig. 5e), liver necrosis was alleviated (Fig. 5f) and serum ALT and AST levels decreased (Fig. 5g). Notably, the number of CD169^+^ macrophages decreased after IFNγ neutralization (Fig. 5h). Next, BMDMs were induced in vitro and then stimulated with IFNγ (Fig. 5i). We observed that almost all of these BMDMs were CD169 positive (Supplementary Fig. 7a), and IFNγ stimulation increased the mean fluorescence intensity of CD169 in the CD11b^+^F4/80^+^ population (Fig. 5j). Peripheral blood mononuclear cells (PBMCs) from HCs were used for further investigation (Fig. 5k and Supplementary Fig. 7b). We found that CD14^+^CD169^+^ monocytes were significantly induced in vitro after 24 h of IFNγ stimulation (Fig. 5l, m).

In summary, CD169^+^ macrophages are IFNγ responsive and IFNγ can induce the expression of CD169 in macrophages and monocytes in vitro.

### CD169^+^ macrophages selectively express CCL12 in AIH

**Fig. 6:**
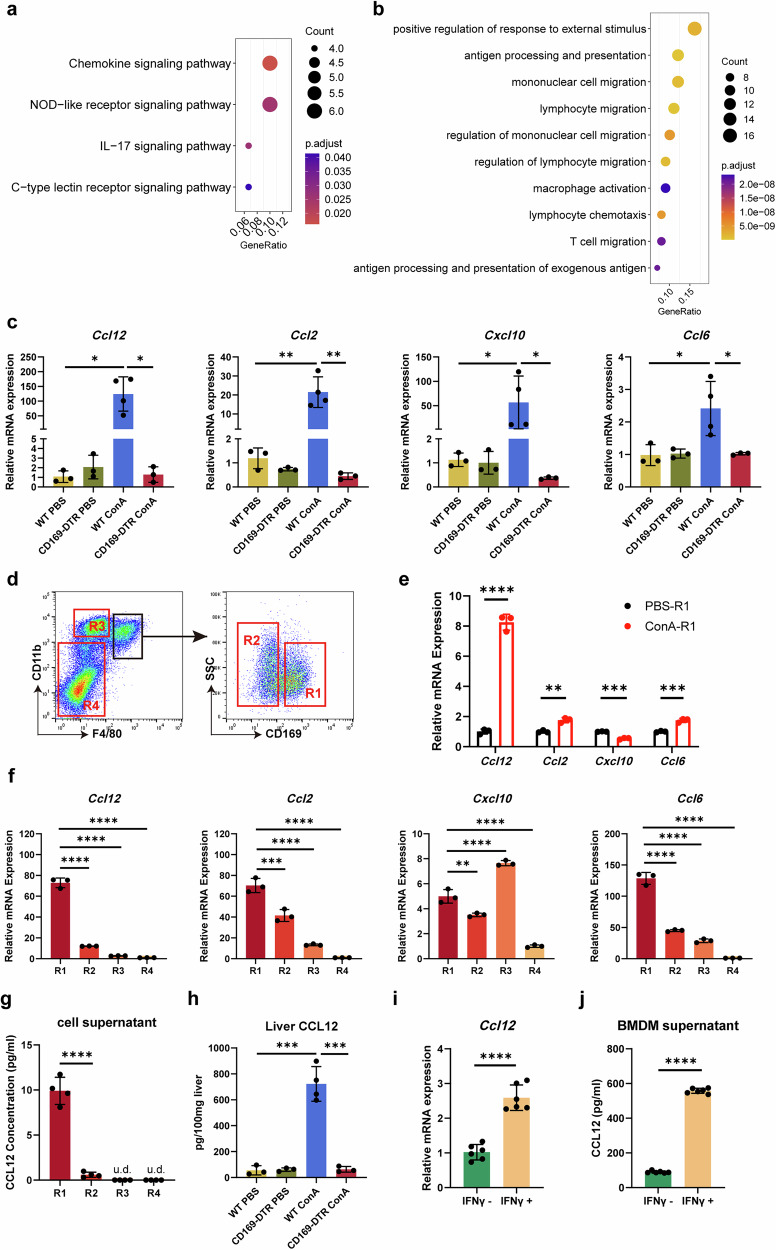
Selective expression of CCL12 in CD169^+^ macrophages in AIH. **a** KEGG analysis of the upregulated genes enriched in the siglec1-positive macrophage subset. **b** GO analysis of the upregulated genes enriched in the siglec1-positive macrophage subset. **c** Relative mRNA levels of *Ccl12*, *Ccl2*, *Cxcl10* and *Ccl6*. **d** Gating strategies for flow cytometry sorting. **e** Relative *Ccl12*, *Ccl2*, *Cxcl10* and *Ccl6* mRNA levels in the sorted CD169^+^ macrophages. **f** Relative *Ccl12*, *Ccl2*, *Cxcl10* and *Ccl6* mRNA levels in the sorted cells from the four gates. **g** Concentrations of CCL12 in the supernatants of the sorted cells from the four gates after culturing 24 h in vitro without any stimulation. **h** Concentrations of CCL12 in the liver homogenate. **i** Relative *Ccl12* mRNA levels of the BMDM with or without stimulation with IFNγ in vitro. **j** Concentrations of CCL12 in the supernatants of the BMDM with or without stimulation with IFNγ in vitro. Results are expressed as mean ± standard error. u.d., under detection. *P < 0.05, **P < 0.01, ***P < 0.001, ****P < 0.0001.

Then, Kyoto Encyclopedia of Genes and Genomes (KEGG) enrichment analysis and Gene Ontology (GO) enrichment analysis were performed to further confirm the characteristics of CD169^+^ macrophages. KEGG enrichment analysis suggested that the chemokine signaling pathway was upregulated in the CD169^+^ macrophages (Fig. 6a). GO enrichment analysis indicated that the pathways of mononuclear cell migration and lymphocyte migration were enriched in CD169^+^ macrophages (Fig. 6b). Chemokine genes associated with these pathways were further identified as *Ccl12*, *Ccl2*, *Cxcl10* and *Ccl6* (Supplementary Fig. 8a). The mRNA levels of these four chemokines increased after ConA administration and significantly decreased after the depletion of CD169^+^ macrophages (Fig. 6c). To further investigate the association between these chemokines and CD169^+^ macrophages, we sorted CD169^+^ macrophages in the livers of WT mice treated with ConA. Also, we reconfirmed that the sorted CD169^+^ cells belong to the CD11b^+^F4/80^+^ subset (Supplementary Fig. 8b). Four cell populations were isolated: R1 (CD11b^+^F4/80^+^CD169^+^), R2 (CD11b^+^F4/80^+^CD169^−^), R3 (CD11b^hi^F4/80^lo^) and R4 (CD11b^neg-lo^F4/80^−^) as shown in Fig. 6d. The purity of the sorted CD169^+^ macrophages in the four different populations was confirmed by qRT–PCR (Supplementary Fig. 8c,d). After ConA administration, the *Ccl12* expressing capacity of CD169^+^ macrophages in the liver was significantly enhanced (Fig. 6e). In addition, *Ccl12* expression levels were significantly increased in the R1 population compared with the R2 population (6.02-fold rise on average, compared with 1.69-fold rise in *Ccl2*, 1.42-fold rise in *Cxcl10* and 2.85-fold rise in *Ccl6*) (Fig. 6f). These results suggest that, compared with *Ccl2*, *Cxcl10* and *Ccl6*, CD169^+^ macrophages are most likely to realize their primary function in AIH by secreting CCL12. A significant increase in CCL12 protein secretion was also detected in the supernatant of the R1 population (average 16.58-fold rise compared with the R2 population) (Fig. 6g). Meanwhile, CCL12 was not detected in the supernatant of R3 or R4 populations, which was consistent with scRNA-seq results (Supplementary Fig. 8e), indicating that CCL12 is expressed in macrophages. In line with our qRT–PCR results (Fig. 6c), scRNA-seq also showed an increase in *Ccl12* expression after ConA administration (Supplementary Fig. 8f). We also measured CCL12 protein levels in mouse liver homogenate (Fig. 6h) and serum (Supplementary Fig. 8g) and found that CCL12 increased after ConA administration and decreased after CD169^+^ macrophage depletion.

Meanwhile, given that CD169^+^ macrophages are IFNγ-responsive, as mentioned above, we investigated whether IFNγ could promote CCL12 expression in CD169^+^ macrophages. We found that BMDMs from WT mice stimulated by IFNγ expressed higher mRNA levels of *Ccl12* (Fig. 6i), and the protein levels of CCL12 in the cell supernatant was also significantly increased (Fig. 6j). In addition, the mRNA levels of the other three chemokines in these BMDMs were examined. *Ccl2* and *Cxcl10*, known IFNγ-responsive chemokines, were upregulated following IFNγ stimulation (Supplementary Fig. 8h).

Taken together, CD169^+^ macrophages actively produce CCL12 in AIH in response to IFNγ stimulation.

### CCL12 recruits monocytes and macrophages to exacerbate AIH

**Fig. 7:**
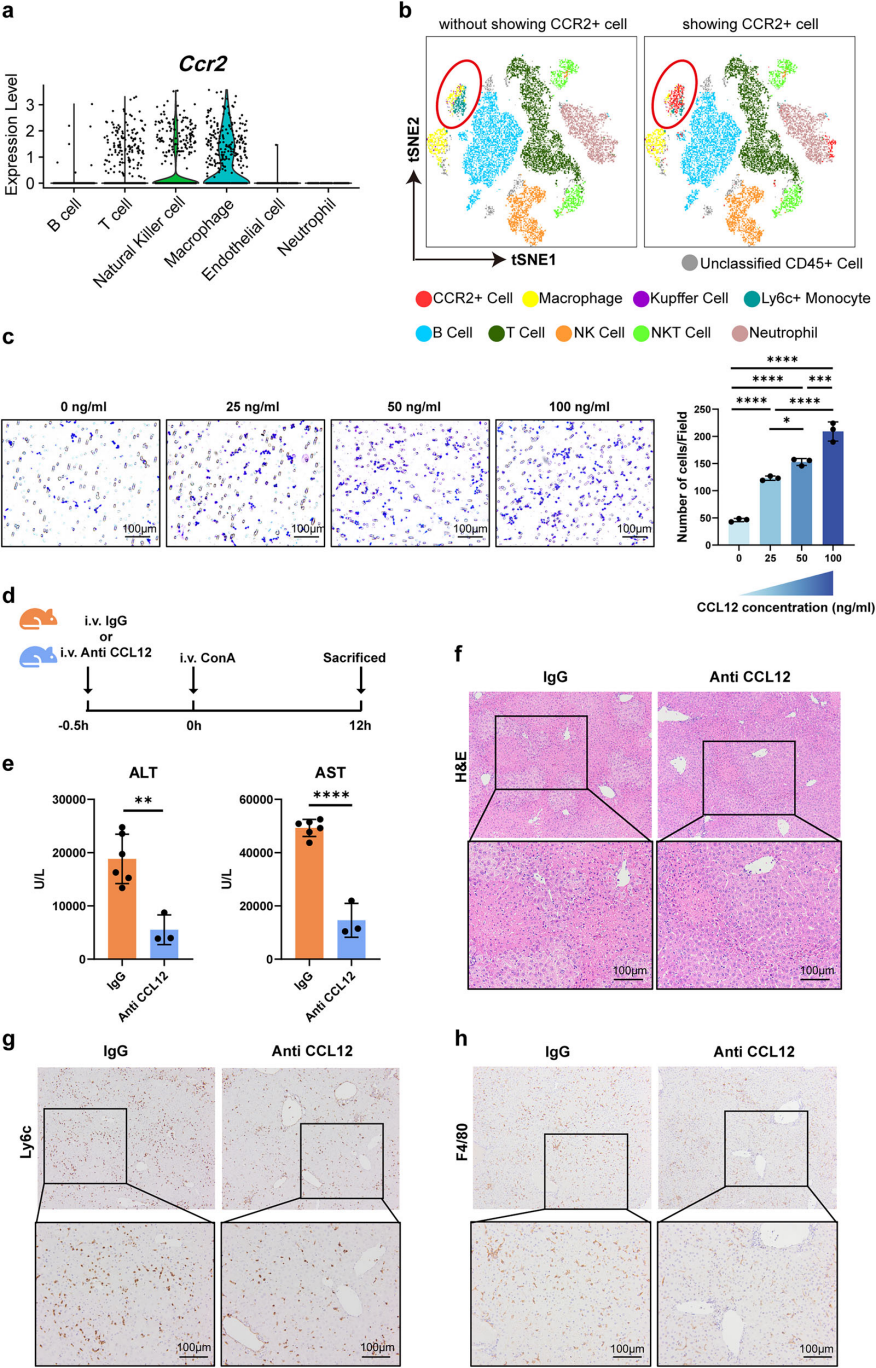
CCL12 recruits monocytes and macrophages, and neutralizing CCL12 improves AIH. **a** Expression of Ccr2 in the different clusters in AIH livers identified by the scRNA-seq dataset. **b** Expression of CCR2 in the different immune cells in the liver of AIH mice identified by flow cytometry. **c** CCL12 chemotaxis RAW264.7 cell line in vitro. **d** Schematic diagram of CCL12 neutralization. Anti-mouse CCL12 antibody and isotype control were both used at 1 μg/g bodyweight intravenously. To induce AIH, 10 mg/kg ConA was injected into mice intravenously. **e** Serum ALT and AST levels of the AIH mice treated with CCL12 neutralizing or isotype antibodies. **f** H&E staining of the AIH mice treated with CCL12 neutralizing antibodies or isotype control. **g** Immunohistochemical staining of Ly6C in the liver sections of the AIH mice treated with CCL12 neutralizing antibody or isotype control. **h** Immunohistochemical staining of F4/80 in the liver sections of the AIH mice treated with CCL12 neutralizing antibody or isotype control. Results are expressed as mean ± standard error. *P < 0.05, **P < 0.01, ***P < 0.001, ****P < 0.0001.

Next, we explored which types of immune cell were chemotactic by CCL12. As CCL12 has only one known receptor, chemokine (C–C motif) receptor 2 (CCR2), we examined CCR2 expression on immune cells in the AIH liver. The results of the scRNA-seq analysis indicated that CCR2 was mainly expressed in macrophages (Fig. 7a). Then, we classified CD45^+^ cells in the liver of AIH using flow cytometry (Supplementary Fig. 9a). After *t*-distributed stochastic neighbor embedding (tSNE) dimensionality reduction processing, we found that the majority of CCR2^+^ cells were Ly6C^+^ monocytes and macrophages (Fig. 7b). This is consistent with our previous results: the infiltration of Ly6C^+^ monocytes and macrophages in the AIH liver decreased after CD169^+^ macrophage depletion (Fig. 4f, g). Therefore, the migration of RAW264.7 cells (a mouse macrophage cell line) toward CCL12 was examined in vitro. We found that the chemotactic effect increased with rising CCL12 concentrations (Fig. 7c). Furthermore, we found that using CCL12 neutralizing antibodies (Fig. 7d) notably improved ConA-induced liver necrosis in mice and reduced serum ALT and AST levels (Fig. 7e, f). Both monocytes and macrophages significantly decreased after neutralizing CCL12, as demonstrated by immunohistochemistry (Fig. 7g, h).

In summary, CCL12 recruited CCR2^+^ monocytes and macrophages, thereby exacerbating AIH in mice.

## Discussion

Previous studies have extensively reported the important role of macrophages in AIH^8,9^, and here we identified that CD169^+^ macrophages are a key subset of them. Compared with the CD169^−^ macrophages in AIH, CD169^+^ macrophages are more responsive to IFNγ and secrete CCL12 more actively, thus recruiting CCR2^+^ monocytes and macrophages as a ‘macrophage amplifier’.

Our results suggest that there are at least two different populations of CD169^+^ macrophages in the liver of AIH: bone marrow-derived Ly6C^hi^ infiltrating macrophages and tissue-resident CD169^+^ macrophages. CD169 has previously been used as a marker of tissue-resident macrophages in many studies^31,32^, but in recent years, an increasing number of studies have shown that CD169^+^ macrophages can also be inflammatory infiltrating macrophages^33,34^. Our results indicated that bone marrow-derived CD169^+^ macrophages are the key macrophage subset in the progression of ConA, suggesting that, in addition to tissue-resident KCs, non-tissue-resident macrophages can also play an important role in acute liver injury.

After the depletion of CD169^+^ macrophages, not only was monocyte and macrophage infiltration in the liver reduced, but T cell infiltration was also decreased. This may be explained by the fact that CXCL10, a chemokine that recruits T cells, is expressed at higher levels in CD169^+^ macrophages, consistent with a previous study^33^. Meanwhile, previous studies have demonstrated that CD169^+^ macrophages can serve as antigen-presenting cells for CTL priming^35^ and promote the early activation of CD8^+^ T cells^36^. Our results suggest that CD169^+^ macrophage depletion has no significant effect on CD8^+^ T cell activation. These findings suggest that the interaction between CD169^+^ macrophages and T cells may be different under various pathological conditions, which may be related to the different expression of surface receptors on T cells under various pathological conditions. In addition, depletion of CD169^+^ macrophages induces a significant expansion of immunosuppressive T_reg_ cells, which contributes to the attenuation of dysregulated autoimmune responses characteristic of AIH, thereby ameliorating the disease progression. Similar results, in which the proportion of T_reg_ cells increased after depletion of CD169^+^ macrophages, have also been reported in other autoimmune diseases^37^, suggesting that CD169^+^ macrophages play an important role in immunosuppression.

CD169 was previously reported to be a type I IFN-inducible protein^26^. Recent studies have shown that IFNγ can induce CD169 on monocytes from patients with Graves’ disease, and the inducing effect is stronger than that of type I IFN (including IFNα and IFNβ)^38^. Our results suggested that CD169^+^ macrophages in AIH were IFNγ responsive.

CCL12, also known as monocyte chemoattractant protein (MCP) 5, has only one receptor, CCR2. In previous studies, CCL12 was found to be involved in inflammation and injury, such as participating in allergic inflammation^39^, promoting bone loss during acute lung injury^40^ and aggravating brain injury in aged mice^41^. In the present study, we found that the CCL12 level was elevated in AIH, suggesting that CCL12 also plays a role in ConA-induced liver injury. Moreover, our results showed that CD169^+^ macrophages could actively produce CCL12 in AIH, and the production of CCL12 in CD169^+^ macrophages could be enhanced by IFNγ in vitro, further illustrating the possible role of CCL12 in inflammatory diseases. However, in contrast to our present findings, a previous study indicated that CD169^+^ macrophages promote colitis by secreting CCL8 rather than CCL12^34^. These results suggest that the chemokine secretion profile of CD169^+^ macrophages can vary across diseases. The immune microenvironment varies across diseases and tissues, causing CD169^+^ macrophages to exhibit different responses and suggesting their flexibility or adaptability in distinct microenvironments.

We acknowledge that our study has some limitations. First, we did not explore the role of CD169^+^ KCs in AIH. Although bone marrow-derived CD169^+^ macrophages play a crucial role in AIH, it is undeniable that the depletion of CD169^+^ KCs may also contribute to the alleviation of acute liver injury. Second, considering that CD169 contained a short cytoplasmic tail that lacks signaling motifs, we addressed the function of CD169^+^ macrophages but not the role of CD169 molecule in AIH. In future studies, we plan to use CD169-specific knockout mice to investigate the function of CD169 and further characterize CD169^+^ macrophages. Third, considering the strong correlation between CD169^+^ macrophages and progression of inflammation in the AIH liver, we used a ConA model to mimic the acute inflammation of AIH and did not explore the role of CD169^+^ macrophages in the chronic progression of AIH, which may not reproduce all clinical characteristics of human disease. Furthermore, CD169^+^ macrophages also play a role in proresolving and reparative responses after an injury^42^. However, post-injury repair is not addressed in our study. In summary, the roles and functions of CD169^+^ macrophages should be further investigated for developing reliable strategies toward AIH.

In summary, we found that CD169^+^ macrophages infiltrate the livers of both mice and humans with AIH, and that bone marrow-derived CD169^+^ macrophages represent a crucial subset driving AIH progression. Depletion of CD169^+^ macrophages ameliorated ConA-induced AIH. In response to IFNγ signaling, CD169^+^ macrophages act like a macrophage amplifier to actively secrete CCL12 to recruit more CCR2^+^ monocytes and macrophages, further aggravating acute liver injury. In the future, targeting bone marrow-derived CD169^+^ macrophages may be a potential strategy for the treatment of AIH.

## Supplementary information

Supplementary Information

## Data Availability

The data are available from the corresponding author on reasonable request.
