# Supplementary material for: Bone marrow-derived CD169+ macrophages promote autoimmune hepatitis by recruiting CCR2+ monocytes via secreting CCL12
Source: Exp Mol Med. 2025 Dec 22;57(12):2930–42. doi: 10.1038/s12276-025-01607-w (PMC12800274; doi:10.1038/s12276-025-01607-w)
Supplement: Supplementary file 1 — Supplementary Information [file 12276_2025_1607_MOESM1_ESM.pdf]

Supplementary Materials

Bone marrow-derived CD169$^+$ macrophages promote autoimmune hepatitis by recruiting CCR2$^+$ monocytes via secreting CCL12

Bingru Lin[1,*], Huayang Zhang[2,*], Pengwei Zhu[3,*], Jianing Chen[4], Dingwu Li[1], Jiaming Zhou[1], Tiantian Zhang[1], Qingxia Chen[1], Chenxi Tang[1], Xin Song[1], Hang Zeng[1], Jinghua Wang[1], Jie Zhang[1], Zhengrui You[2,#], Xiong Ma[2,#], Chaohui Yu[1,#]

Supplementary Methods

Human participants and samples

The Scheuer scoring system was applied to evaluate the inflammation grades and fibrosis stages. The whole blood from HCs was obtained, and peripheral blood mononuclear cells (PBMCs) were later separated for flow cytometry analysis. All individuals were enrolled in Shanghai Renji Hospital and provided written informed consent. The study was carried out under the principles of the Declarations of Helsinki and Istanbul and approved by the Shanghai Jiao Tong University Ethics Committee.

Mice and treatments

All mice were housed in a special pathogens-free facility (20-24℃) with a 12h/12h light/dark cycle. ConA (Sigma, C2010) was dissolved in sterile PBS and injected into mice through the tail vein. The dosage and modeling time were indicated in each experiment. Three consecutive days of 10μg/kg DT (sigma, D0564) intraperitoneal administration was used to deplete CD169$^+$ cells in CD169-DTR mice, and wild-type

(WT) mice received the same treatment for control. For interferon (IFN) γ neutralizing, anti-mouse IFNγ antibody (Bioxcell, BE0055) was used at 25μg/g bodyweight intravenously, and isotype control (Bioxcell, BE0088) was used at the same dosage. For CCL12 neutralizing, anti-mouse CCL12 antibody (R&D, AF428) was used at 1μg/g bodyweight intravenously, and isotype control (R&D, AB-108-C) was used at the same dosage. Thirty minutes after the administration of neutralizing antibodies, 10mg/kg ConA was injected into mice to induce AIH. The mice were sacrificed 12 hours later. Liver tissues were fixed with 4% paraformaldehyde for further histological staining. The animal study was approved by the Animal Care and Use Committee of the First Affiliated Hospital, Zhejiang University School of Medicine.

PBMC isolation and stimulation

Briefly, removing plasma by centrifugation, then diluting blood cells with an equal volume of phosphate-buffered saline (PBS) and adding them to Ficoll (GE Healthcare) with an equal volume of blood cells. PBMCs were aspirated after centrifugation and washed twice with PBS. Next, PBMCs were resuspended in RPMI 1640 medium (with 10% fetal bovine serum (FBS), 1% penicillin/streptomycin, and 50mmol/L 2-Mercaptoethanol) and then spread into 24-well plates at a concentration of 5*10^5/ml. The cells were then incubated in a cell incubator with or without 30ng/ml IFNγ (PeproTech) for 24 hours before flow cytometry.

Serum biochemical analysis

Blood obtained from the vein was allowed to stand for half an hour at room temperature before centrifugation at 3000rpm for 10 minutes. Then, the supernatant serum was

collected. Alanine aminotransferase (ALT) and aspartate aminotransferase (AST) were analyzed by automatic biochemical analyzer LW C400 (Lanyun Medical Technology) using corresponding kits (ALT01, AST01, Purebio Biotechnology) according to the manufacturer's instructions.

Chemokine and cytokine assays

The serum collected was used for IFNγ, tumor necrosis factor (TNF)α, and CCL12 enzyme-linked immunosorbent assay (ELISA) according to the kit instructions. IFNγ and TNFα ELISA kit were purchased from proteintech (KE10001 and KE10002). CCL12 ELISA was provided by R&D (MCC120). The mouse liver was made into 10% homogenate using physiological saline for CCL12 detection, and the final data was converted to the content of CCL12 per milligram of the liver.

RNA extraction and quantitative real-time PCR

Total RNA from liver tissues or cells was extracted by trizol. The concentrations of RNA were unified and further reverse transcribed to cDNA with the Evo M-MLV RT Premix Kit (Accurate Biotechnology, AG11706). SYBR Green Premix Pro Taq HS qPCR Kit (Accurate Biotechnology, AG11701) was used for qPCR procedures in ABI QuantStudio5DX (Thermo). The relative expression levels of target genes were normalized to gapdh or β-actin calculated by the $2^{-\Delta\Delta CT}$ method. The primer sequences are listed in Supplementary Table 2.

Histological staining

Paraffin liver sections were deparaffinized and rehydrated. Then hematoxylin-eosin staining was performed to verify the necrosis area in the livers. As for

immunohistochemistry or immunofluorescence, the sections were incubated in 3% hydrogen peroxide to block endogenous peroxidase, and then heat epitope retrieval was performed. Then the sections were incubated with primary antibody overnight at 4℃. After incubation with corresponding secondary antibodies on the second day, the DAB kit (ZSGB-BIO, ZLI-9018) was used to detect the positive staining in immunohistochemistry. The primary antibodies for immunohistochemistry listed as below: anti-CD19 (Abcam, ab245235), anti-CD3 (Abcam, ab215212), anti-NKp46 (Abcam, ab233558), anti-MPO (CST, 15178), anti-Ly6C (Abcam, ab314120), anti-F4/80 (CST, 70076), anti-mouse CD169 (Abcam, ab312840), anti-human CD169 (Abcam, ab183356); The secondary antibody for immunohistochemistry: ZSGB-BIO, PV-6001. The primary antibodies for immunofluorescence are listed below: anti-CD169 (Biorad, MCA884), anti-F4/80 (CST, 70076), and anti-CLEC4F (R&D, AF2784); The secondary antibody for immunofluorescence: Donkey anti Rat AF647 (Abcam, ab150155), Donkey anti Goat AF488 (Abcam, ab150129), Goat anti Rat AF647 (Abcam, ab150159), Goat anti-Rabbit AF594 (Thermo, A-11012). TUNEL staining was operated according to the kit instructions (Beyotime, C1086).

Cell lines and migration assay

RAW264.7 cell line was purchased from the Chinese Academy of Science and cultured in Dulbecco's modified Eagle's medium containing 10% FBS and 1% penicillin/streptomycin. The cells were incubated at 37℃ in a 5% CO_2 incubator. The chemotactic capacity of CCL12 was valued using an 8-μm-pore transwell system (LABSELECT). Briefly, the same number of RAW264.7 cells was seeded in the upper

chamber in serum-free medium and the lower chambers were applied with 20% FBS and gradient concentrations of CCL12 (MCE, HY-P7246). The migration cells in the lower chamber were counted under the microscope after 24 hours.

Bone marrow-derived macrophages (BMDM) preparation and stimulation

The femur and tibia bones of 6-week-old C57BL/6 mice were isolated and rinsed with a 1 mL syringe in RPMI 1640 medium. Then the cells passed through a 70μm cell strainer and the red blood cells were lysed. Next, the cells were resuspended in RPMI 1640 medium with 10% FBS, 1% penicillin/streptomycin, and 40ng/ml M-CSF (novoprotein). The medium was renewed on day 3. On day 6, the medium was changed as M-CSF was removed and 100ng/ml IFNγ (PeproTech) was added. After stimulating for 24 hours, the BMDMs were gently scraped off for flow cytometry analysis or applied with trizol for total RNA extraction. And the supernatant was also collected for CCL12 detection.

Bone marrow transplantation and genotyping

The bone marrow of WT or CD169-DTR recipients was destroyed by a 7.5 Gary γ-ray radiation. 6 hours later, bone marrow cells obtained (as mentioned above) from healthy WT mice were injected into the radiated WT or CD169-DTR mice. Bone marrow cells obtained from CD169-DTR mice were injected into the radiated CD169-DTR mice. Each of the radiated mice received 10 million new bone marrow cells. 4 weeks after the transplantation, peripheral blood was collected and genotyping was performed to verify the efficiency of bone marrow reconstitution. Then, the mice were injected with 20mg/kg ConA and were sacrificed 24 hours later.

It is worth explaining that conventional bone marrow transplantation experiments typically include four experimental groups. In this study, we omitted the group transplanting bone marrow cells from CD169-DTR mice into WT mice. This decision was made because bone marrow transplantation cannot completely replace 100% of the recipient's bone marrow cells with donor cells. As shown in Supplementary Fig. 3c, the transplanted mice become chimeras containing both donor-derived bone marrow cells and recipient's original bone marrow cells. When transplanting WT mice bone marrow (whose CD169 cells do not express DTR) into CD169-DTR mice followed by DT injection, this approach preserves CD169 cells originating from WT donor bone marrow while eliminating $CD169^+$ macrophages derived from both the bone marrow and liver-resident populations of CD169-DTR recipients. However, transplanting CD169-DTR bone marrow into WT recipients followed by DT injection would leave the WT recipients with their original $CD169^+$ macrophages from both bone marrow-derived and liver-resident populations, making it impossible to distinguish between the two origins of macrophages. Therefore, we excluded the group transplanting CD169-DTR bone marrow cells into WT mice.

Single-cell RNA sequencing analysis

Data in GSE124395 and GSE201006 was downloaded and analyzed. The DoubletFinder R package was used to screen suitable cells, and then the filtered data was normalized. The Seurat v4.0.1 R package was used for data integration. After principal component analysis, the data was visualized by t-distributed stochastic neighbor embedding (t-SNE). The expression level of genes was visualized through the

"Featureplot" and "Vlnplot" functions in the Seurat R package. Then we performed gene enrichment analyses using the clusterProfiler R package. The enriched genes upregulated in the two clusters were identified using "FindMarkers", and the top 100 differentiated genes were used for subsequent gene enrichment analysis. HALLMARK, KEGG, and GO gene enrichment analyses were used in this study.

Isolation of hepatic non-parenchymal cells

The mice were drained of blood. Livers were then removed, minced into 3-5mm pieces, and resuspended in 100 U/ml collagenase IV (Gibco) and 100 μg/ml DNase (Sigma). Followed by shaking in a 37 ℃ shaker at 150 rpm for 1 hour, the cells were passed through a 70μm cell filter, and 36% percoll was used to isolate liver nonparenchymal cells. After removing the upper layer of hepatocytes, the red blood cells in the bottom layer were lysed. Finally, these cells were then used for flow cytometry analysis or cell sorting.

Flow cytometry

The resuspended hepatic non-parenchymal cells of mice were stained with a Zombie Aqua Fixable Viability Kit (Biolegend, 423102) and then blocked by anti-mouse CD16/32 (BD, 553142). For staining of surface biomarkers, the following antibodies were used: APC-CY7 anti-mouse CD45 (30-F11, BD, 557659), FITC anti-mouse CD11b (M1/70, Biolegend, 101205), BV421 anti-mouse F4/80 (T45-2342, BD, 565411), BV605 anti-mouse TIM-4 (RMT4-54, BD, 745206), PE-CY7 anti-mouse Ly6C (AL-21, BD, 560593), BV786 anti-mouse Ly6G (1A8, BD, 740953), BV650 anti-mouse CCR2 (475301, BD, 747968), PE anti-mouse CD169 (3D6.112, Biolegend,

142404), AF700 anti-mouse CD3e (500A2, BD, 557984), APC anti-mouse B220 (RA3-6B2, BD, 553092), PE-CF594 anti-mouse NK1.1 (PK136, BD, 562864), PE-CY7 anti-mouse CD3e (145-2C11, BD, 552774), BV421 anti-mouse CD4 (GK1.5, BD, 562891), PE-CY5.5 anti-mouse CD8a (53-6.7, BD, 551162), APC anti-mouse CD62L (MEL-14, BD, 553152), FITC anti-mouse CD44 (IM7, BD, 561859), BV785 anti-mouse CD25 (PC61, Biolegend, 102051), and PE anti-mouse Ly6C (AL-21, BD, 560592). For the intracellular staining, cells were harvested and stimulated with a leukocyte activation cocktail (BD, 550583) for 6 hours at 37 °C before using a transcription factor buffer set (BD, 562574). Then cells were stained with FITC anti-mouse IFNγ (XMG1.2, BD, 554411), BV605 anti-mouse IL4 (11B11, BD, 564007), APC anti-mouse IL17A (TC11-18H10.1, BD, 506916), and PE anti-mouse FOXP3 (MF23, BD, 560414).

The PBMCs from human samples were stained with fixable viability stain 780 (BD, 565388), PE anti-human CD169 (7-239, BD, 565248), PE-CY7 anti-human CD11b (ICRF44, BD, 557743), APC anti-human HLA-DR (G46-6, BD, 560744), BV421 anti-human CD33 (WM53, BD, 562854), BV510 anti-human CD14 (MΦP9, BD, 563079), BV605 anti-human CD15 (W6D3, BD, 562980).

Stained cells were assessed on Cytoflex LX (Beckman) or Fortessa X-20 (BD). Data were analyzed with FlowJo v10.

Cell sorting and culture

The hepatic non-parenchymal cells of mice obtained above were stained with fixable viability stain 780 (BD, 565388) and then blocked by anti-mouse CD16/32. The cells were stained by FITC anti-mouse CD11b (M1/70, Biolegend, 101205), BV421 anti-

mouse F4/80 (T45-2342, BD, 565411), and PE anti-mouse CD169 (3D6.112, Biolegend, 142404), and then sorted by flow cytometry sorting system MoFlo (Beckman). The sorted cells were treated with trizol immediately for qPCR analysis or cultured in RPMI 1640 medium for supernatant collection. Briefly, the sorted cells were counted and seeded into 96-well plates with 10000 cells per well. The supernatant was collected 24 hours later for CCL12 detection.

Statistical analysis

Data were presented as the mean ± standard error and analyzed by GraphPad Prism 8 software. Student's t-test or one-way ANOVA was conducted for statistical analysis as appropriate. The correlation between biochemical indicators and the number of positive CD169 cells in AIH patients was analyzed using Spearman's correlation coefficient. All analyses were two-tailed and P values < 0.05 were considered statistically significant.

Supplementary Figures

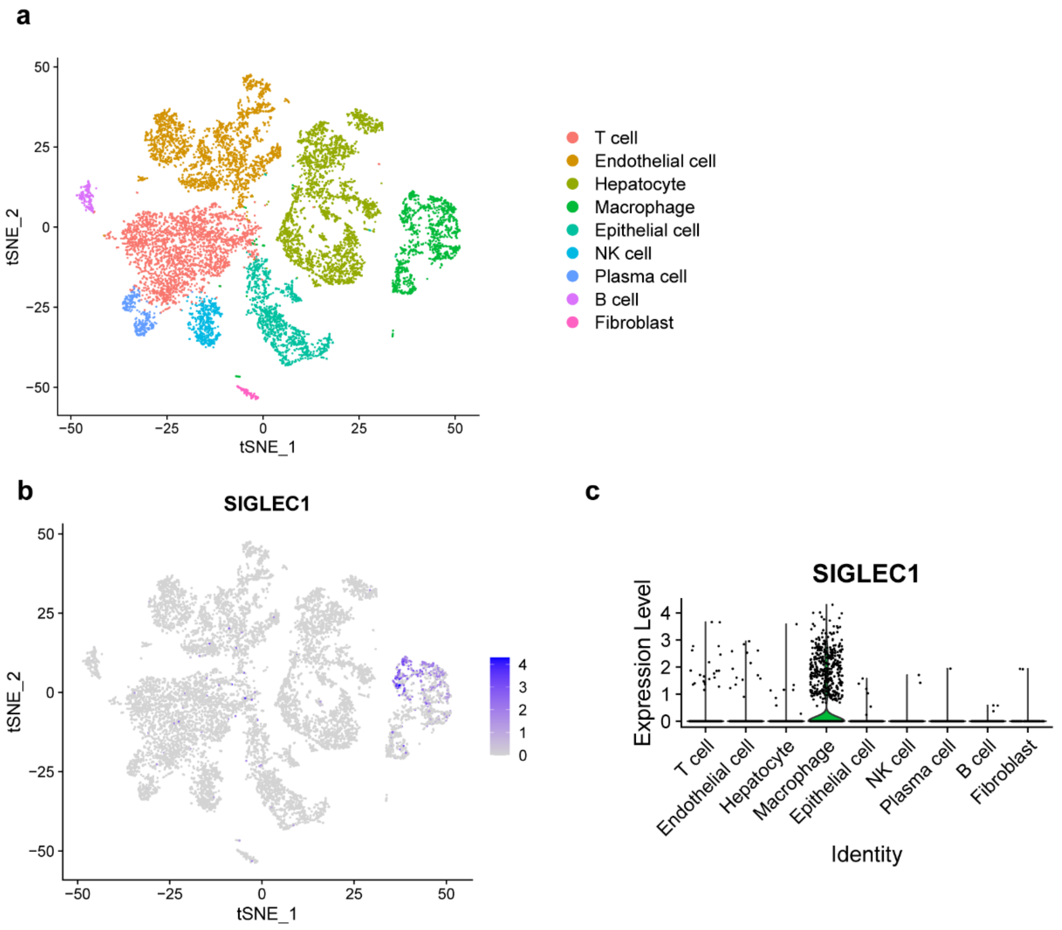

Supplementary Fig. 1 Single-cell RNA sequencing in human liver from GSE124395 dataset showed that siglec1 (the gene encoding CD169) expressed in macrophages.

(a) Identification of cell clusters from the GSE124395 dataset.

(b) Expression of siglec1 in the different cell clusters.

(c) Siglec1 distribution in different cell clusters.

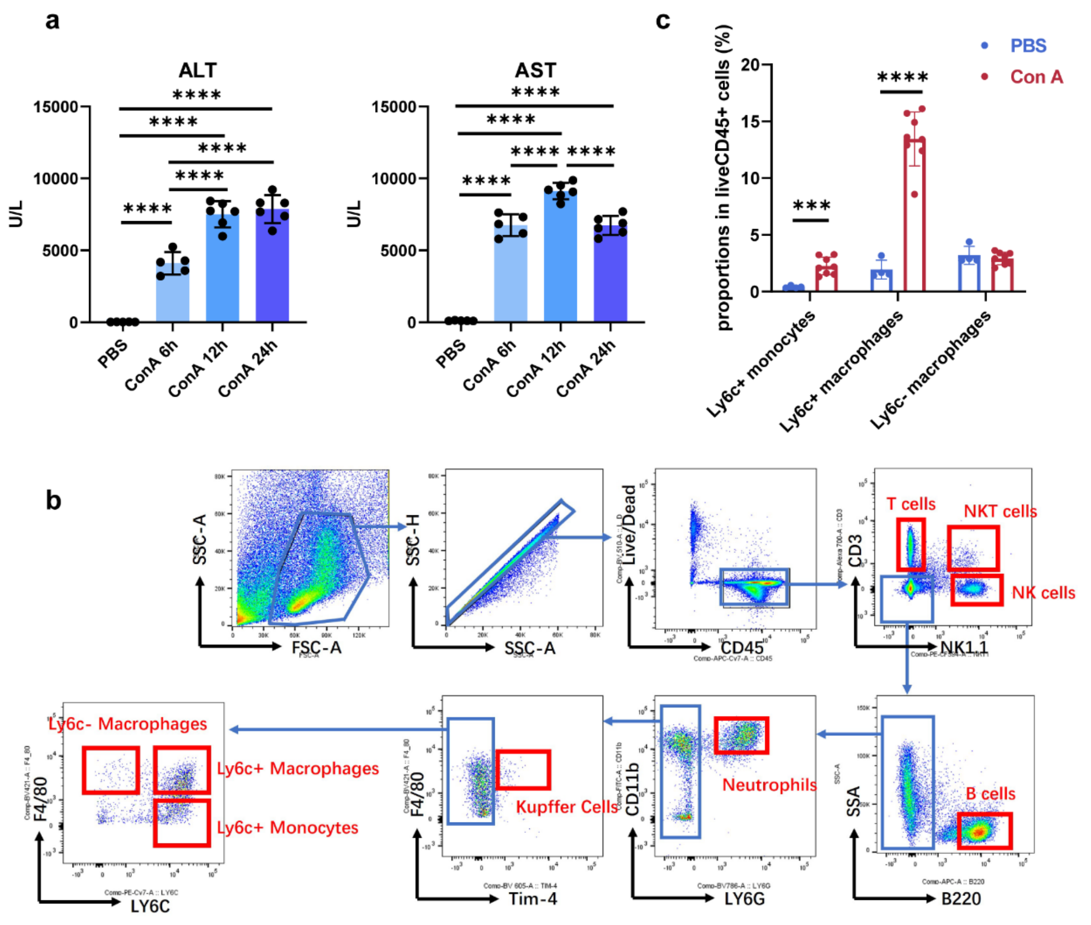

Supplementary Fig. 2 ALT, AST and immune cells changed after ConA administration.

(a) ALT and AST levels at indicated time points after ConA administration.

(b) Gating strategies in Fig.2.

(c) Proportions of monocytes and macrophages after ConA administration.

Results are expressed as mean ± standard error. *p<0.05, **p<0.01, ***p<0.001, ****p<0.0001.

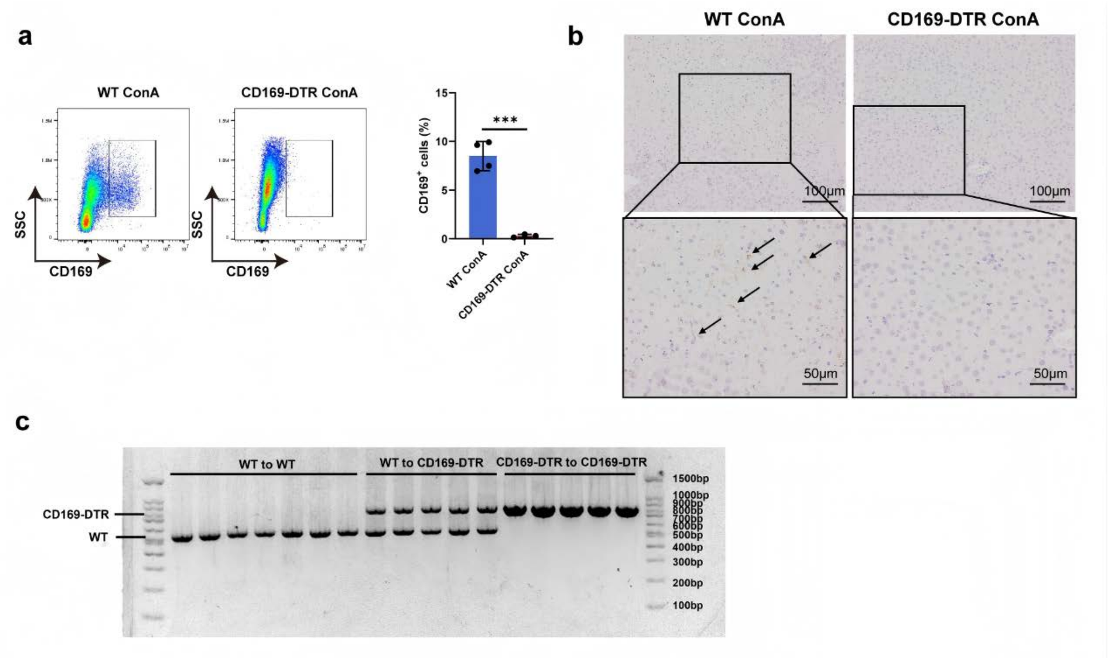

Supplementary Fig. 3 Confirmation of CD169^{+} cells depletion and bone marrow transplantation.

(a) Verification of CD169^{+} cells depletion by flow cytometry.

(b) Verification of CD169^{+} cells depletion by immunohistochemical staining of CD169.

(c) Verification of the efficiency of bone marrow transplantation by genotyping.

Results are expressed as mean ± standard error. *p<0.05, **p<0.01, ***p<0.001, ****p<0.0001.

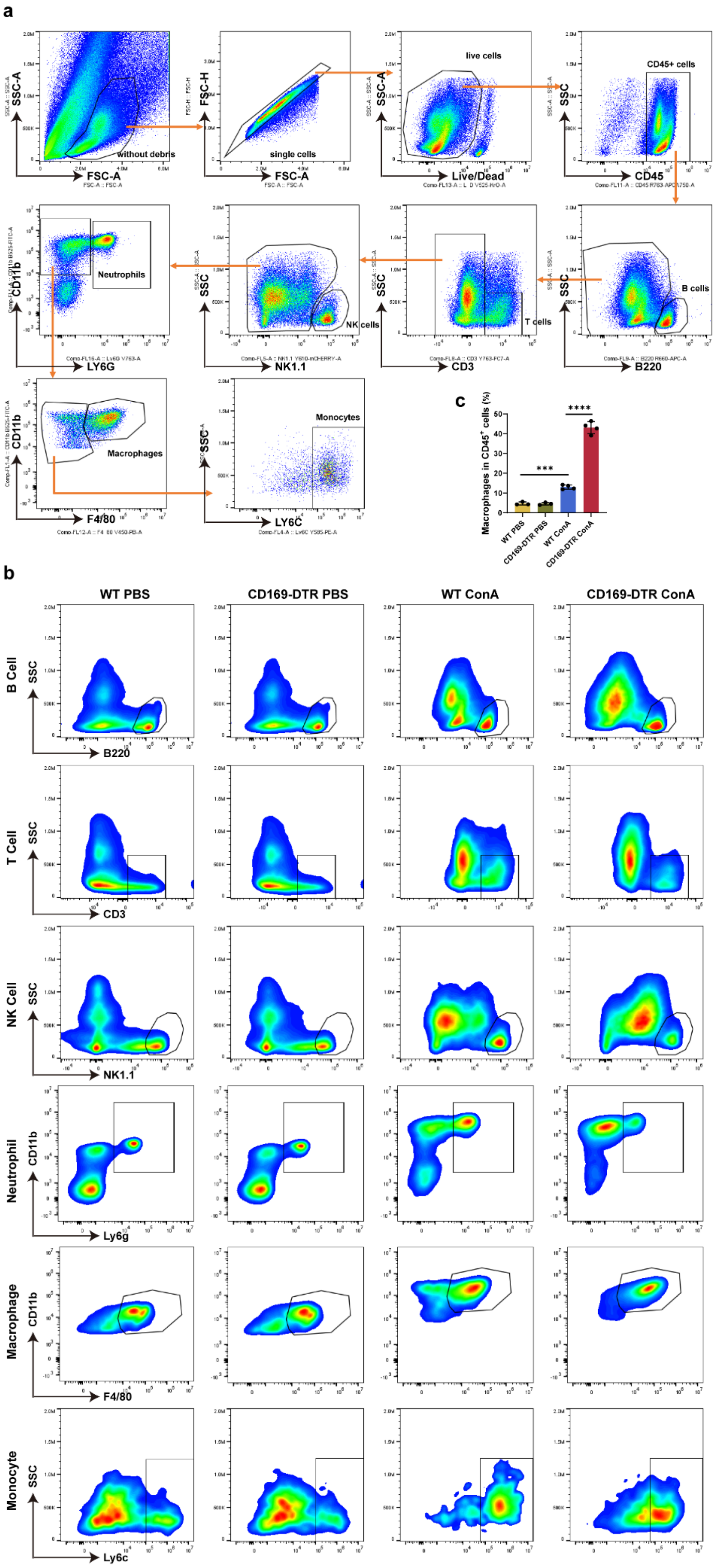
a
SSC-A
FSC-A
without debris
FSC-H
single cells
live cells
Live/Dead
SSC
CD45
CD45+ cells
CD11b
LY6G
Neutrophils
NK1.1
NK cells
CD3
T cells
B220
B cells
F4/80
Macrophages
LY6C
Monocytes
c
Macrophages in CD45+ cells (%)
WT PBS
CD169-DTR PBS
WT ConA
CD169-DTR ConA
***
****
b
WT PBS
CD169-DTR PBS
WT ConA
CD169-DTR ConA
B Cell
T Cell
NK Cell
Neutrophil
Macrophage
Monocyte
Ly6g
Ly6c

Supplementary Fig. 4 CD169$^+$ macrophages depletion altered the frequency of various immune cells in the liver.

(a) Gating strategies.

(b) Representative plots of B cells, NK cells, T cells, Neutrophils, Ly6C$^+$ monocytes, and macrophages in CD45$^+$ cells in the four groups.

(c) Frequency of macrophages in the four groups.

Results are expressed as mean ± standard error. *p<0.05, **p<0.01, ***p<0.001, ****p<0.0001.

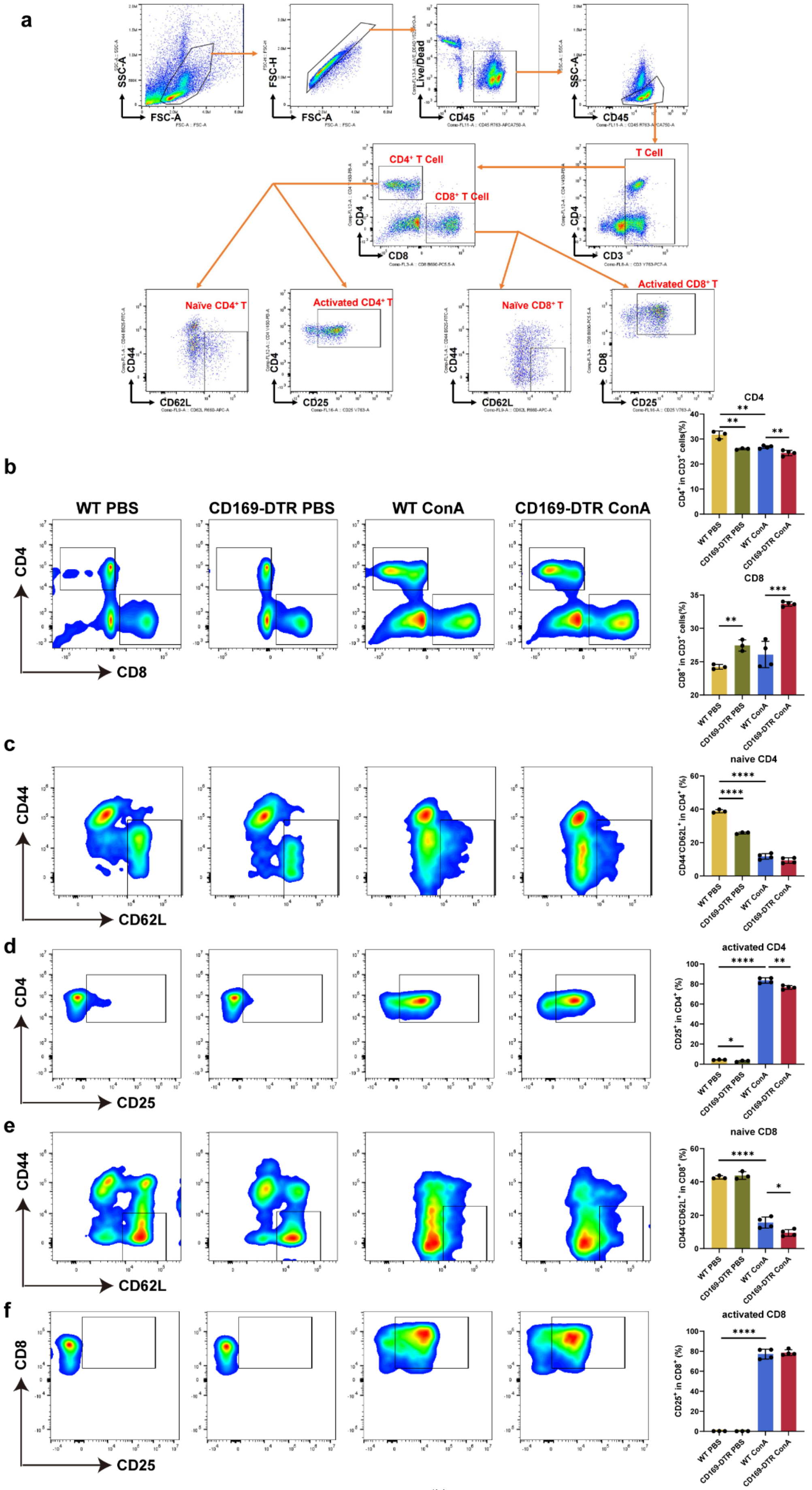
a
SSC-A
FSC-A
FSC-H
Live/Dead
CD45
T Cell
CD4
CD3
CD4⁺ T Cell
CD8⁺ T Cell
CD8
Naïve CD4⁺ T
Activated CD4⁺ T
Naïve CD8⁺ T
Activated CD8⁺ T
CD44
CD62L
CD25
b
WT PBS
CD169-DTR PBS
WT ConA
CD169-DTR ConA
CD4
CD4⁺ in CD3⁺ cells(%)
CD8⁺ in CD3⁺ cells(%)
c
naive CD4
CD44⁻CD62L⁺ in CD4⁺ (%)
d
activated CD4
CD25⁺ in CD4⁺ (%)
e
naive CD8
CD44⁻CD62L⁺ in CD8⁺ (%)
f
activated CD8
CD25⁺ in CD8⁺ (%)

Supplementary Fig. 5 CD169$^+$ macrophages depletion altered the activation of T cells in the liver.

(a) Gating strategies.
(b) Frequency of CD4$^+$ T cells and CD8$^+$ T cells in the CD3$^+$ T cells.
(c) Frequency of CD62L$^+$ CD44$^-$ naïve CD4$^+$ T cells in the CD4$^+$ T cells.
(d) Frequency of CD25$^+$ activated CD4$^+$ T cells in the CD4$^+$ T cells.
(e) Frequency of CD62L$^+$ CD44$^-$ naïve CD8$^+$ T cells in the CD8$^+$ T cells.
(f) Frequency of CD25$^+$ activated CD8$^+$ T cells in the CD8$^+$ T cells.

Results are expressed as mean ± standard error. *p<0.05, **p<0.01, ***p<0.001, ****p<0.0001.

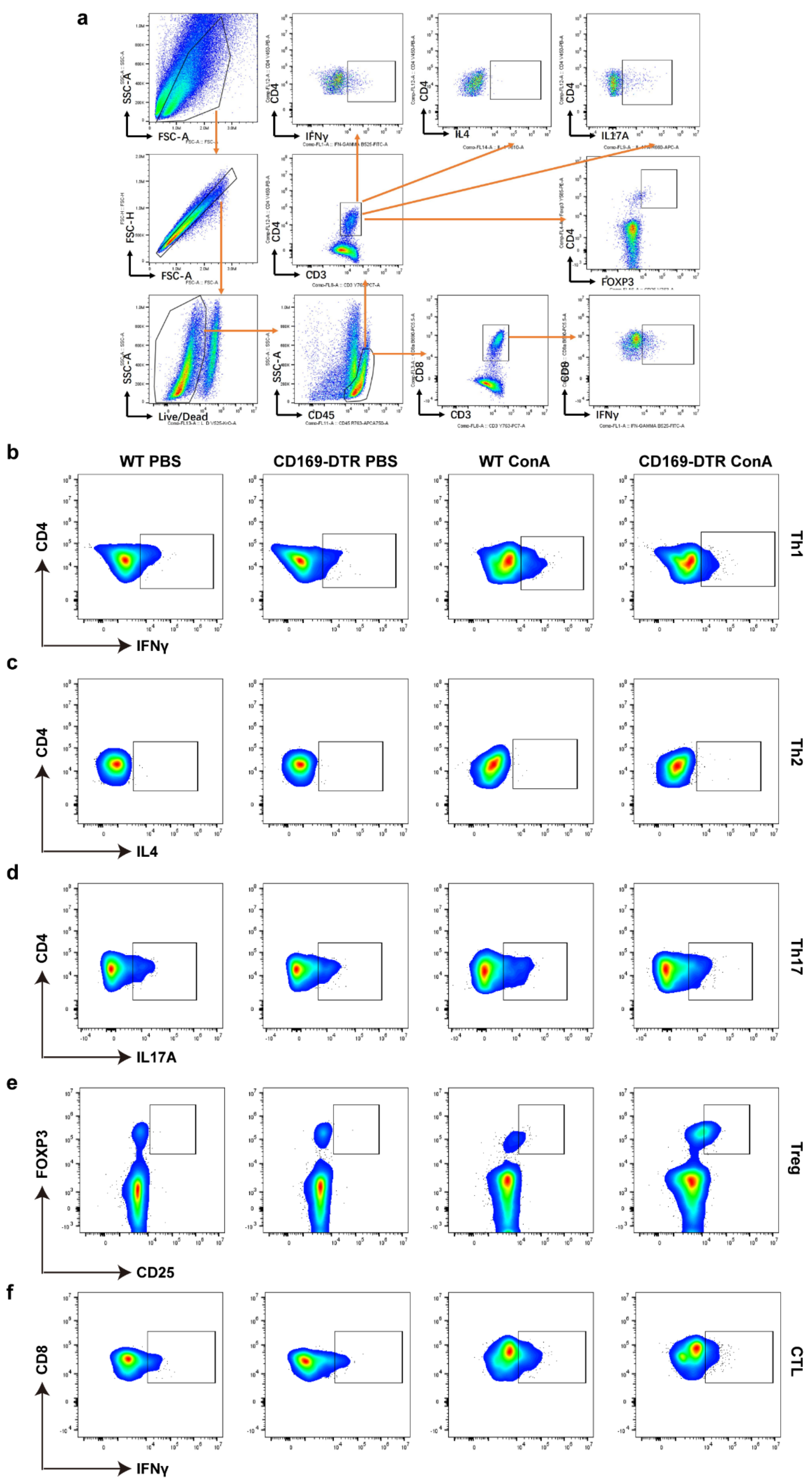
a
SSC-A
FSC-A
FSC-H
Live/Dead
CD45
CD4
CD3
IFNγ
IL4
IL17A
FOXP3
CD8
b
WT PBS
CD169-DTR PBS
WT ConA
CD169-DTR ConA
Th1
c
Th2
d
Th17
e
CD25
Treg
f
CTL

Supplementary Fig. 6 CD169^{+} macrophages depletion altered the differentiation of T cells in the liver.

(a) Gating strategies.
(b) Representative plots of IFNγ^{+} Th1 cells in the CD4^{+} T cells.
(c) Representative plots of IL4^{+} Th2 cells in the CD4^{+} T cells.
(d) Representative plots of IL17A^{+} Th17 cells in the CD4^{+} T cells.
(e) Representative plots of FOXP3^{+} CD25^{+} Treg cells in the CD4^{+} T cells.
(f) Representative plots of IFNγ^{+} CTL cells in the CD8^{+} T cells.

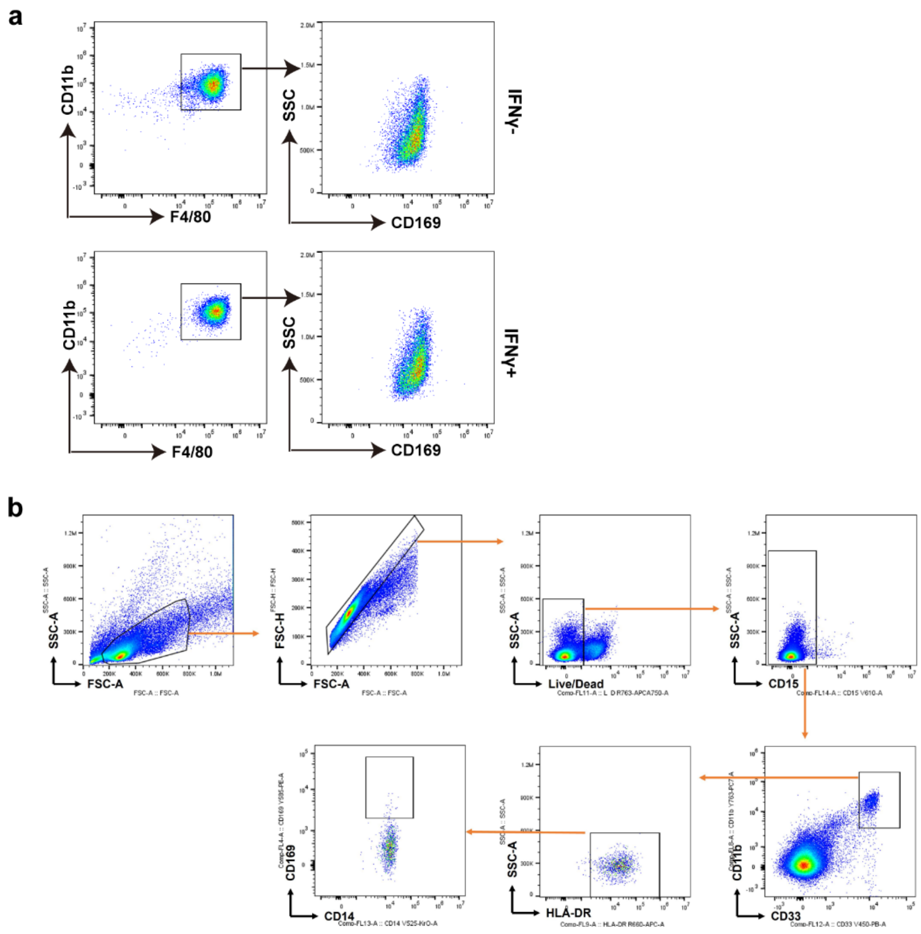

Supplementary Fig. 7 CD169 can be induced by IFNγ.

(a) Representative plots of CD11b$^+$F4/80$^+$CD169$^+$ cells in the BMDMs.

(b) Gating strategies for CD169$^+$ monocytes in human PBMC.

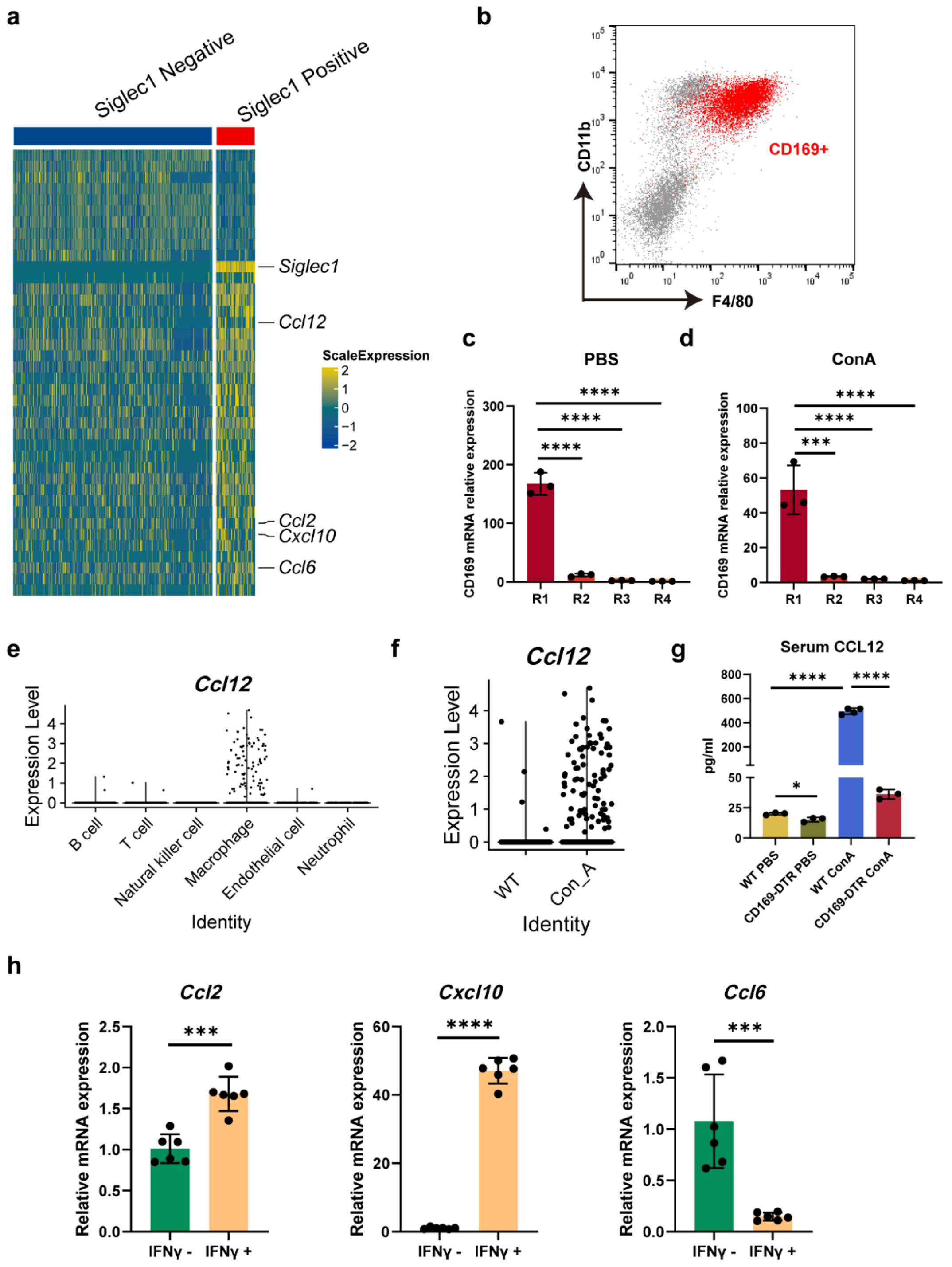

Supplementary Fig. 8 CD169^{+} macrophages sorting confirmation and CCL12 distribution.

(a) Top chemokines in the Siglec1 positive subset.

(b) Representative plots of CD169^{+} cells distribution.

(c) Relative CD169 mRNA levels normalized by Gapdh in the sorted cells in PBS mice.

(d) Relative CD169 mRNA levels normalized by Gapdh in the sorted cells in ConA mice.

(e) CCL12 distribution in different cell clusters in the liver of AIH mice identified by single-cell RNA sequencing.

(f) CCL12 expression increased in the ConA group identified by single-cell RNA sequencing.
(g) Concentrations of serum CCL12 in the mice.
(h) Relative Ccl2, Cxcl10, and Ccl6 mRNA levels normalized by Gapdh in the bone marrow-derived macrophages with or without stimulation with 100ng/ml IFNγ for 24 hours in vitro.

Results are expressed as mean ± standard error. *p<0.05, **p<0.01, ***p<0.001, ****p<0.0001.

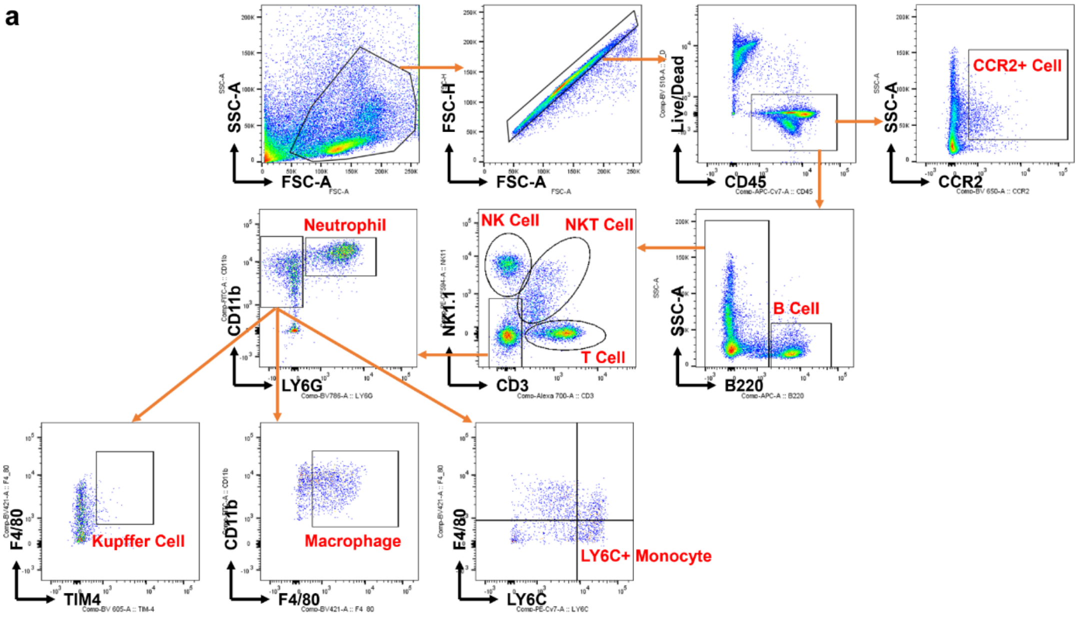

Supplementary Fig. 9 Gating strategies in Fig.7b

Supplementary Table 1 Comparison of clinical characteristics of enrolled healthy controls and patients with AIH, CHB, PBC and MASLD.

	HC(n=6)	AIH(n=40)	CHB(n=7)	PBC(n=21)	MASLD(n=12)
Age (years)	33.0±6.6	46.7±12.4	45.7±10.2	47.5±10.6	45.3±13.0
Gender (F/M)	3/3	31/9	2/5	20/1	6/6
ALT (U/L)	26.5(21.3,35.5)	72.5(14,282.1)	45.3(26.0,57.0)	29.0(18.0,61.0)	37.5(20.5,83.0)
AST (U/L)	26.0(23.5,27.0)	38.5(18.5,214.5)	29.0(23.0,38.0)	29.0(19.0,46.0)	22.0(19.8,47.2)
ALP (U/L)	95.5(84.5,110.3)	69.5(55.8,109.5)	81.0(77.0,111.0)	127.0(72.0,218.0)	77.5(71.8,81.5)
γ-GT (U/L)	43.0(19.3,50.3)	38.0(14.8,103.5)	20.0(17.0,30.5)	89.0(29.0,123.0)	38.5(24.0,75.3)
TBIL (μmol/L)	8.6(7.8,10.2)	15.7(9.3,22.3)	17.2(8.15,18.7)	10.4(8.1,14.1)	10.3(8.8,12.0)
IgG (g/L)	NA	15(11.9,17.7)	14.3(12.7,17.3)	13.7(12.3,15.0)	12.6(11.3,13.6)

Ages were shown as mean±standard error. Serum biochemical indicators were shown as median (25% percentile, 75% percentile).

HC: healthy control; AIH: autoimmune hepatitis; CHB: chronic hepatitis B; PBC: primary biliary cholangitis; MASLD: metabolic dysfunction-associated steatotic liver disease; F/M: female/male; ALT: alanine aminotransferase; AST: aspartate transaminase; ALP: alkaline phosphatase; γ-GT: γ-glutamyl transferase; TBIL: total bilirubin; IgG: immunoglobulin G.

Supplementary Table 2 Primers for qPCR

Target Name	Primer Sequence 5'-3'
Ifnγ	forward- CTGGCAGGATGATTCTGCTGG
	reverse-GCATACGACAGGGTTCAAGTTAT
Tnfα	forward- CCCTCACACTCAGATCATCTTCT
	reverse-GCTACGACGTGGGCTACAG
IL6	forward- TAGTCCTTCCTACCCCAATTTCC
	reverse-TTGGTCCTTAGCCACTCCTTC
IL1β	forward- GCAACTGTTCCTGAACTCAACT
	reverse-ATCTTTTGGGGTCCGTCAACT
Ccl12	forward- ATTTCCACACTTCTATGCCTCCT
	reverse-ATCCAGTATGGTCCTGAAGATCA
Ccl2	forward- TTAAAAACCTGGATCGGAACCAA
	reverse-GCATTAGCTTCAGATTTACGGGT
Cxcl10	forward- CCAAGTGCTGCCGTCATTTTC
	reverse-GGCTCGCAGGGATGATTTCAA
Ccl6	forward- GCTGGCCTCATACAAGAAATGG
	reverse-GCTTAGGCACCTCTGAACTCTC
Cd169	forward- AGTGATAGCAACCGCTGGTTA
	reverse-GCACAGGTAGGGTGTGGAAC
Gapdh	forward- AGGTCGGTGTGAACGGATTTG
	reverse-TGTAGACCATGTAGTTGAGGTCA
β-Actin	forward- GGCTGTATTCCCCTCCATCG
	reverse-CCAGTTGGTAACAATGCCATGT
